# Design, Synthesis, Computational Studies, and Anti-Proliferative Evaluation of Novel Ethacrynic Acid Derivatives Containing Nitrogen Heterocycle, Urea, and Thiourea Moieties as Anticancer Agents

**DOI:** 10.3390/molecules29071437

**Published:** 2024-03-23

**Authors:** Abdelmoula El Abbouchi, Khaoula Mkhayar, Souad Elkhattabi, Nabil El Brahmi, Marie-Aude Hiebel, Jérôme Bignon, Gérald Guillaumet, Franck Suzenet, Saïd El Kazzouli

**Affiliations:** 1 Euromed Research Center, Euromed Faculty of Pharmacy, School of Engineering in Biomedical and Biotechnology, Euromed University of Fes (UEMF), Meknes Road, Fez 30000, Morocco; a.elabbouchi@ueuromed.org (A.E.A.); n.elbrahmi@ueuromed.org (N.E.B.); 2 Institut de Chimie Organique et Analytique, Université d’Orléans, UMR CNRS 7311, BP 6759, CEDEX 2, 45067 Orléans, France; marie-aude.hiebel@univ-orleans.fr (M.-A.H.); franck.suzenet@univ-orleans.fr (F.S.); 3 Laboratory of Engineering, Systems and Applications, National School of Applied Sciences, Sidi Mohamed Ben Abdellah-Fez University, Fez 30040, Morocco; khaoula.mkhayar@usmba.ac.ma (K.M.); souad.elkhattabi@usmba.ac.ma (S.E.); 4 Institut de Chimie des Substances Naturelles, CNRS, Université Paris-Saclay, 91190 Gif-sur-Yvette, France; jerome.bignon@cnrs.fr

**Keywords:** ethacrynic acid, nitrogen heterocycle, urea, thiourea, anti-proliferative, GSTP1-1, caspase 3, drug-likeness, ADMET properties, molecular docking

## Abstract

In the present work, the synthesis of new ethacrynic acid (**EA**) derivatives containing nitrogen heterocyclic, urea, or thiourea moieties via efficient and practical synthetic procedures was reported. The synthesised compounds were screened for their anti-proliferative activity against two different cancer cell lines, namely, HL60 (promyelocytic leukaemia) and HCT116 (human colon carcinoma). The results of the in vitro tests reveal that compounds **1**–**3**, **10**, **16**(**a**–**c**), and **17** exhibit potent anti-proliferative activity against the HL60 cell line, with values of the percentage of cell viability ranging from 20 to 35% at 1 μM of the drug and IC_50_ values between 2.37 μM and 0.86 μM. Compounds 2 and 10 showed a very interesting anti-proliferative activity of 28 and 48% at 1 μM, respectively, against HCT116. Two **PyTAP**-based fluorescent **EA** analogues were also synthesised and tested, showing good anti-proliferative activity. A test on the drug-likeness properties in silico of all the synthetised compounds was performed in order to understand the mechanism of action of the most active compounds. A molecular docking study was conducted on two human proteins, namely, glutathione S-transferase P1-1 (pdb:2GSS) and caspase-3 (pdb:4AU8) as target enzymes. The docking results show that compounds **2** and **3** exhibit significant binding modes with these enzymes. This finding provides a potential strategy towards developing anticancer agents, and most of the synthesised and newly designed compounds show good drug-like properties.

## 1. Introduction

Cancer is a major health issue that can be expressed as the leading cause of death. However, current cancer treatments can still be improved due to their relatively significant side effects [1]. Thus, the development of new cancer therapies with more specificity and selectivity is very requested [2]. Advanced studies in medicinal chemistry play a pivotal role in addressing the ever-evolving challenges posed by cancer treatment. Despite significant advancements in therapeutic interventions, drug resistance and adverse side effects remain arduous obstacles. Thus, the development of novel anticancer agents with improved efficacy and safety profiles is crucial. One of the most promising targets for the development of anticancer agents is the resistance against existing drugs, which remains a major challenge to overcome [3]. In fact, microsomal glutathione transferase 1 (mGST1) and glutathione transferase pi (GSTpi) are often overexpressed in tumour cells and confer resistance against a number of cytostatic drugs, such as cisplatin and doxorubicin (DOX) [4]. The diuretic drug ethacrynic acid (**EA**) is a potent reversible inhibitor of GSTP1-1 by covalent binding thanks to its α, β-unsaturated carbonyl functional group that binds to the cysteine residue in the active site via a Michael-like addition [5]. **EA** inhibits tumour cell growth at high concentrations and enhances the therapeutic efficiencies of several anticancer agents [6,7,8]. Moreover, some further insights into the mode of action of the **EA** analogues suggested a more complex behaviour than the sole inhibition of GSTpi.

In our previous work, we have demonstrated that **EA** analogues containing amide or triazole induced apoptosis through caspase activation [9,10]. The activation of caspases being one of the effective strategies in the treatment of cancer, this result encouraged us to develop novel anticancer agent analogues of **EA**.

**Figure 1:**
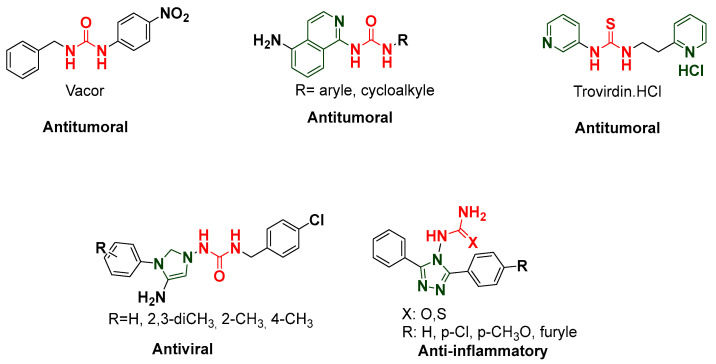
Structures of some biologically active bearing urea and thiourea moieties.

Thiourea and urea are classes of organic compounds, which stand out due to their extensive diversity and versatile applications. Derivatives of these compounds exhibit a wide range of pharmacological activities, encompassing antimicrobial, antidiabetic, analgesic, and anticancer properties [11,12,13,14,15] as illustrated in Figure 1.

In our continuous efforts to develop new anticancer agents based on **EA** [10,16,17,18,19], we wish to report a new **EA** family, which undoubtedly provides new chances to fight against cancer. In this work, we were specifically interested in synthesising novel **EA** derivatives bearing on their carboxylic acid part various heterocyclic, urea, and thiourea moieties. Subsequently, the anti-proliferative activity in vitro against HL60 (promyelocytic leukaemia) and HCT116 (human colon carcinoma) cancer cell lines was evaluated. Simultaneously, two fluorescent **EA** analogues have also been synthetised and characterised according to their anti-proliferative activity. Moreover, we evaluated the drug-likeness properties in silico of all the synthetised compounds. The molecular docking of some selected compounds against two human proteins, namely, glutathione S-transferase P1-1 pocket (pdb:2GSS) and caspase-3 (pdb:4AU8) as target enzymes, was achieved.

## 2. Results and Discussions

### 2.1. Chemistry

**Scheme 1:**
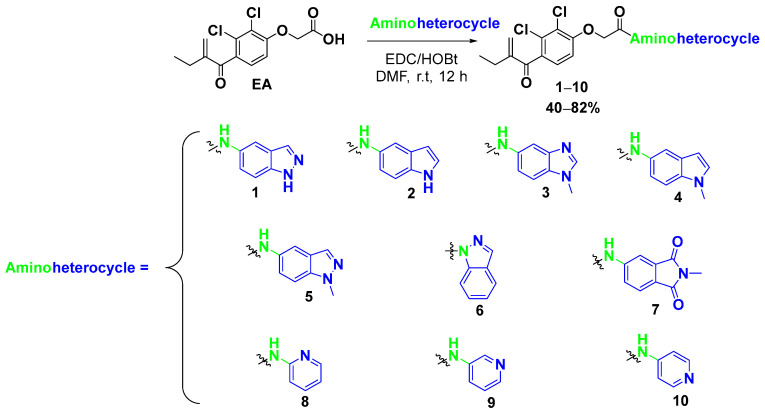
Synthesis of **EA** derivatives (**1**–**10**) containing a nitrogen heterocyclic ring.

Our target compounds containing aminoheterocycles (**1**–**10**) were synthesised according to the reaction pathway described in Scheme 1. The desired heterocyclic motifs were introduced via a general peptide coupling procedure between **EA** and various heterocyclic amines in the presence of EDC/HOBt. The reaction was carried out in DMF for 12 h at room temperature. Flash column chromatography, eluting with different proportions of DCM/EtOAc, gave pure compounds of **EA** derivatives (**1**–**10**) in 40–82% yields.

The insertion of a heterocyclic group bearing a free amine function, as in the cases of aminoindazole and aminoindole, is achieved in reasonable yields of 50 and 62%, respectively. Apparently, the secondary amine is more reactive than the primary amine. These results were confirmed by ^1^H NMR, where we noted signals corresponding to NH but no signal for NH_2_.

**Scheme 2:**
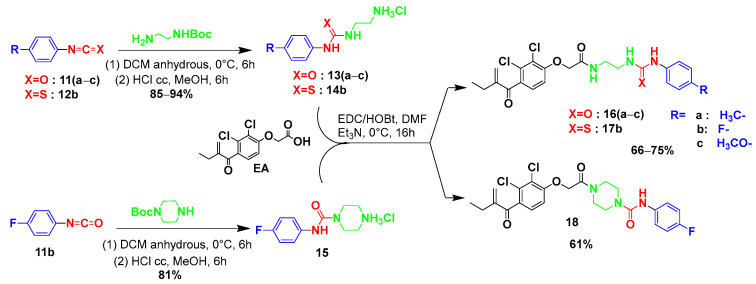
Synthesis of a new urea and thiourea **EA** derivatives **16**(**a**–**c**), **17b**, and **18**.

The importance of urea and thiourea functions in cancer applications, as previously discussed, has allowed us to develop a new class of **EA** analogues bearing a urea or a thiourea function as outlined in Scheme 2. The compounds **16**(**a**–**c**), **17b**, and **18** were synthesised through a two-step sequence employing two types of linkers. The first step involved *N*-Boc-ethylenediamine and *N*-Boc-piperazine with the substituted phenyl isocyanates **11**(**a**–**c**) or the 4-flurophenylisothiocyanate **12b** in dry DCM. The resulting Boc-protected intermediates were then subjected to Boc group cleavage using concentrated HCl in methanol at room temperature giving the desired amines **13**(**a**–**c**), **14b**, and **15** as hydrochloride salts. All intermediates were dried and used directly in the next step without further purification. Afterwards, a conventional peptide synthesis was employed from the intermediates **13**(**a**–**c**), **14b**, or **15** and **EA** in the presence of EDC/HOBt in DMF to afford **EA** derivatives **16**(**a**–**c**), **17b**, and **18** in yields ranging between 61 and 75%.

**Scheme 3:**
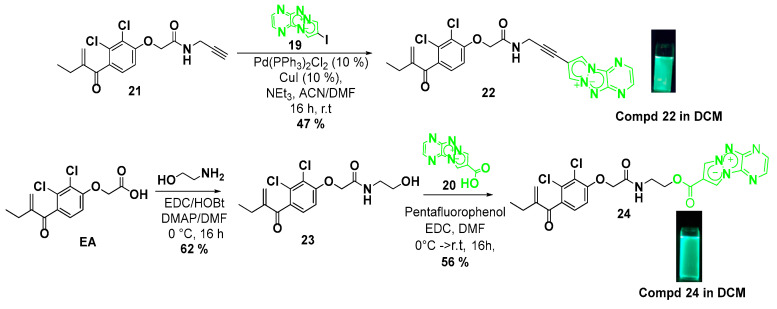
Synthesis of new fluorescent **EA** analogues **22** and **24**.

Subsequently, we have prepared two novel **EA** derivatives modified onto the carboxylic acid part with **PyTAP** fluorescent molecules [20] (Scheme 3). The preparation of the pyridazino-1,3a,6a-triazapentalene (**PyTAP**) pharmacophores **19** and **20** was achieved according to procedures from the literature [21]. Compound **22** was prepared using a Sonogashira-type coupling reaction between compound **19** and **EA** derivative **21** bearing an alkyne group. The reaction was carried out using Pd(PPh_3_)_2_Cl_2_/CuI as a catalyst in the presence of triethylamine as a base in a CH_3_CN/DMF mixture at room temperature for 16 h following our previously reported procedure [19]. This sequence leads to the desired compound **22** in 47% yield. Regarding compound **24**, the synthesis was carried out through a two-step sequence from **EA** according to Scheme 3. First, the intermediate compound **23** was prepared in a satisfactory yield (62%) using the classical condition of peptide coupling from **EA** and 2-aminoethan-1-ol. Then an esterification reaction was performed between the pharmacophore **20** and the compound **23** using pentafluorophenol/EDC as activating agents in DMF at room temperature for 16 h. Under these conditions, compound **24** was obtained with a 56% yield (Scheme 3).

All the synthesised compounds in this study were purified by chromatography eluting with different proportions of DCM/EtOAc and then characterised by ^1^H and ^13^C NMR and IR and HRMS. All products were in full accordance with their depicted structures.

### 2.2. Photophysical Properties

**Figure 2:**
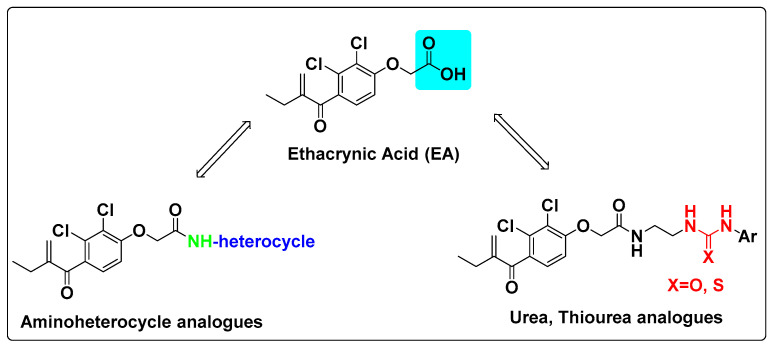
Ethacrynic acid analogue series.

**Figure 3:**
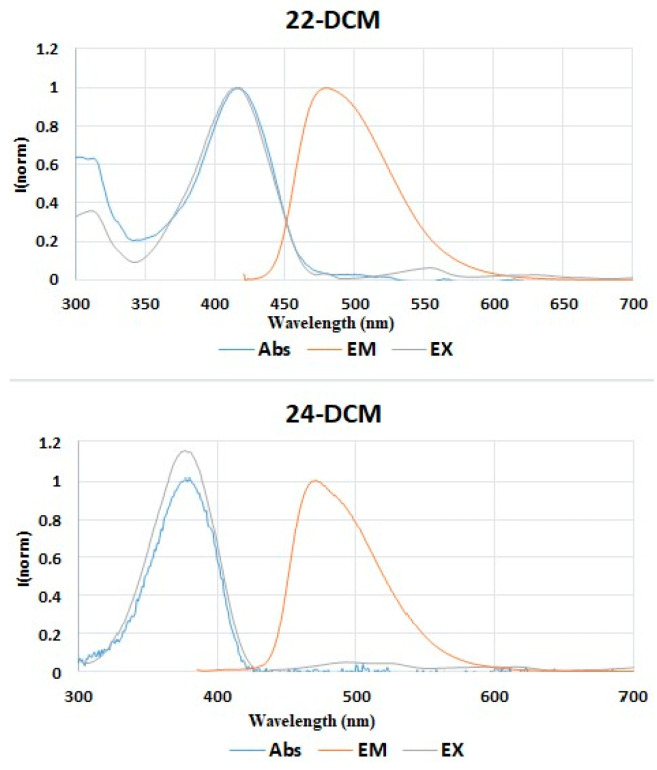
UV-Vis absorption (Abs), excitation (Ex), and emission (Em) spectra of compounds **22** and **24** dispersed in DCM.

As shown in Table 1 and Figure 2, the fluorescence analysis results clearly demonstrate that both analogues **22** and **24** exhibit fluorescence properties. The presence of the ester function on compound **24** allows, on one hand, the quantum yield to be doubled compared to analogue **22** with a value of 0.93, and on the other hand, the Stokes shift value to be improved by 128 nm compared to 68 nm for **22**. However, compound **22** remains brighter with a value of 3584. Both analogues emit in the green range and exhibit interesting spectroscopic properties (Figure 3), allowing us to perform future fluorescence tests on living cells.

### 2.3. Biological Study

**Figure 4:**
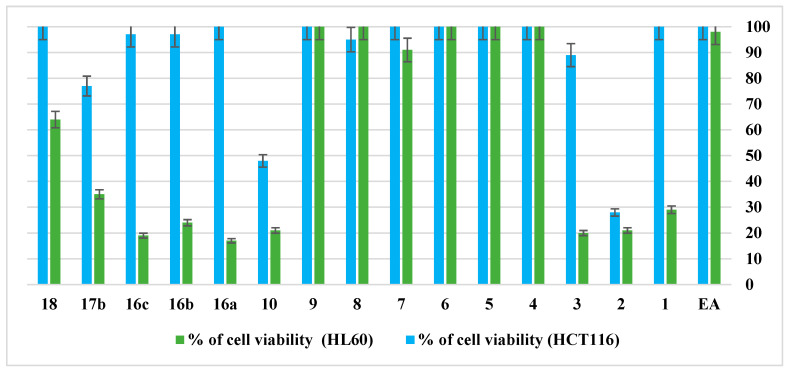
Percentage of viability at 1 μM of **EA** and its derivatives against HL60 and HCT116.

Figure 4 showed the anti-proliferative activities of **EA** used as a control and its analogues containing aminoheterocycles, urea, or thiourea moieties against two different cancer cell lines, namely, HL60 (promyelocytic leukaemia) and HCT116 (human colon carcinoma). The anti-proliferative activities are based on the evaluation of the percentage of cell viability using the sensitive CellTiter-Glo^®^ luminescent assay, which is a homogeneous method to determine the number of viable cells in culture, and it is based on the quantitation of ATP (an indicator of metabolically active cells) [23]. The viability of both HL60 and HCT116 was measured for all the synthesised compounds at 1 μM final concentration.

The results show that eight derivatives of the **EA** including **1**–**3**, **10**, **16**(**a**–**b**), and **17b** exhibit very high anti-proliferative activity at 1 μM against the HL60 cell line, with values of the percentage of cell viability ranging from 20 to 35%. In the case of HCT116, only compounds **2** and **10** are active with values of cell viability of 28 and 48%, respectively. Unfortunately, all the other compounds have shown low anti-proliferative activity on both cancer cell lines at 1 μM. The obtained results display that compounds **1**, **3**, and **16**(**a**–**c**) have a good selectivity towards HL60 over HCT116 cell lines. The structure–activity relationship (SAR) showed that the position of the nitrogen atom within pyridine has a notable influence on the anti-proliferative activity. In fact, when the nitrogen is at position 2 or 3 (compounds **8** and **9**), no activity on the two studied cell lines was observed. Whereas compound **10**, which has a nitrogen molecule at position 4, exhibits interesting activity on both cancer cell lines (HL60 and HCT116). We also noticed that modification of the carboxylic acid part of **EA** by the introduction of urea or thiourea moieties provided anti-proliferative activity similar to that of aminoheterocyclic moieties against HL60 and HCT116 cell lines.

**Figure 5:**
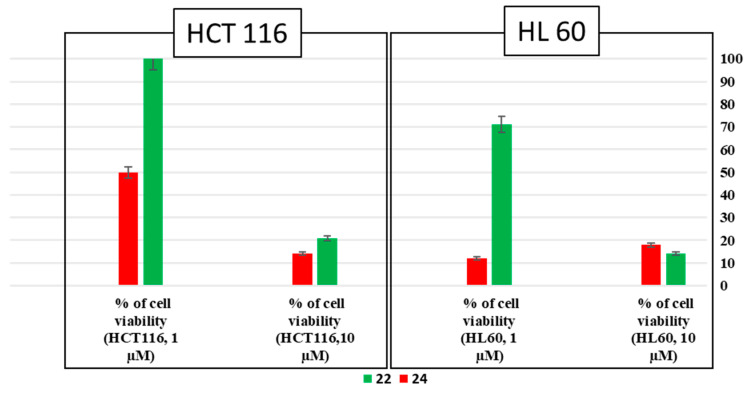
Percentage of anti-proliferative activities of compounds **22** and **24** at two different concentrations (1 µM and 10 µM) against HL60 and HCT116.

Concerning the fluorescent **EA** analogues **22** and **24**, the anti-proliferative activity was also determined against the same cancer cell lines at two different concentrations (1 µM and 10 µM). Compound **22** exhibits activity on both HL60 and HCT116 cell lines at 10 µM with viabilities of 14% and 21%, respectively. When the concentration was decreased to 1 µM, a loss of activity was observed on both cancer cells with viabilities of 71% (HL60) and 100% (HCT116). However, the ester compound **24** is active on both cancer cell lines HL60 and HCT116 at both concentrations, with values of cell viability of 12% and 49% at 1 µM and 18% and 14% at 10 µM, respectively (Figure 5).

The IC_50_ values were determined for all the compounds that induced a reduction in the viability of over 50% on the HL60 cell line. Among the selected **EA** analogues, **1**–**3**, **10**, **16**(**a**–**b**)**, 17b**, and **24** showed a strong anti-proliferative activity against HL60, with IC_50_ values ranging from 0.86 to 6.43 μM (Table 2). Notably, compounds containing a urea moiety, **16a** and **16b**, exhibited similar anti-proliferative activities to those bearing aminoheterocyclic groups **1**–**3** and **10**. Products **16a** and **16b** displayed IC_50_ values of 2.37 μM and 0.86 μM, respectively, against HL60. Conversely, compound **17b**, which features a thiourea group, demonstrated lower activity with an IC50 of 6.43 μM. This decrease in anti-proliferative activity is mainly attributed to the shift from a urea functional group to a thiourea functional group.

### 2.4. Drug-Likeness Prediction

The physicochemical properties and drug-likeness of the synthesised compounds were evaluated, and the relevance of Lipinski’s rule of five [24], Veber’s rule [25], and Egan’s rule [26] was determined. The aim is to ensure that the compounds comply with these filters and meet the necessary criteria for drug-like molecules. Only compounds that meet the criteria without any deviation were selected for the purpose of a docking simulation. In this work, the drug-likeness properties of the synthetised molecules are predicted using the SwissADME online tool (https://www.swissadme.ch/, accessed on 11 February 2024) [27]. Based on the obtained results for the parent **EA** and the analogues **1**–**10**, **16**(**a**–**c**), **17b**, and **18** (Table 3 and Table 4), the log *p* values of the compounds are less than five, meaning that they are all soluble in aqueous solutions. All molecules exhibit both a hydrogen bond donor and acceptor less than 5 and 10, respectively. The number of rotational bonds of molecules is less than 10, except for analogues **16**(**a**–**c**), **17b**, and **18.** All compounds have a molecular weight below 500D (Table 3), except for analogue **18**, indicating that the compounds can easily cross cell membranes [28,29]. In addition, synthetic accessibility, i.e., the ease with which this molecule can be synthesised in the laboratory, was evaluated [30]. In our case, all compounds show synthetic accessibility values below 10, which meet the criteria of a fairly easy access. All molecules exhibit a high absorption regarding their bioavailability score stated as 0.55. This typically means that 55% of the administered substance is absorbed and becomes available to the body’s circulation and to target tissues. To summarise, the molecules (**1**–**10**) meet all criteria for development in medicinal chemistry.

### 2.5. Molecular Docking

**Figure 6:**
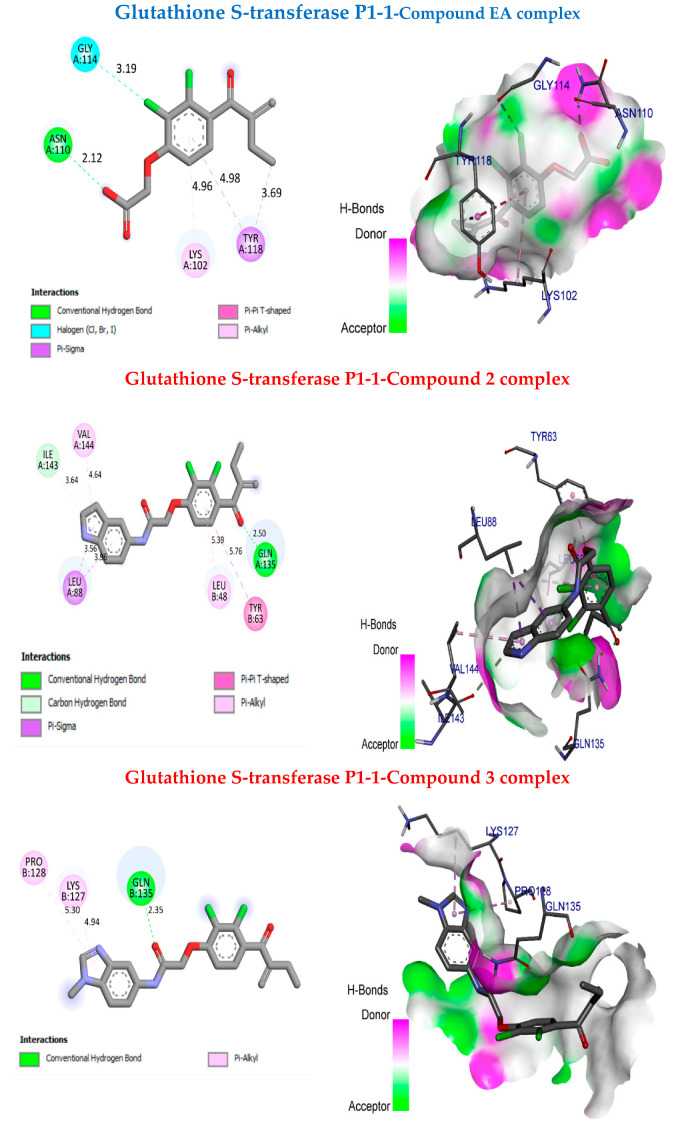
Molecular docking between the molecules **EA**, **2**, and **3** and the human glutathione S-transferase P1-1 pocket.

In this study, molecular docking aims to predict and analyse the binding and interactions of selected molecules through drug-likeness. Compounds **2** and **3** were chosen due to their compatibility with drug-likeness properties, with compound **2** was selected for its activity on HL60 and HCT116 cell lines, and compound **3** was selected for its high biological activity, particularly on the HL60 cell line. A comparison of these interactions is conducted with those established between the reference ligand **EA** and the active site of proteins, human glutathione S-transferase P1-1 (pdb:2GSS), and caspase-3 (pdb:4QU8), obtained from the RCSB Protein Data Bank. Molecular docking simulations were performed using Autodock Vina software (ADT) MGLTools 1.5.6 packages, and the resulting data were analysed using Discovery Studio 2016 Client software. Figure 6 presents the molecular docking for **EA**, **2**, and **3** in the human glutathione S-transferase P1-1 pocket. The binding affinity values of 2 and 3 are −6.7 kcal/mol and 7.0 kcal/mol, respectively. However, the reference compound **EA** has a binding affinity value of −6.2 kcal/mol. Compounds **2** and **3** are more stable in the human glutathione S-transferase P1-1 pocket than **EA** because the binding affinity value of the reference compound is greater than the binding affinity values of the synthesised compounds **2** and **3**. In addition, compound **2** exhibits six interactions: two important hydrogen bonds with ILe A134 and Gln A135 at distances of 3.46 Å and 2.5 Å, respectively, and four hydrophobic bonds with LeuA88, LeuB48, Tyr B63, and Val A144 at distances 3.56 Å, 5.39 Å, 5.76 Å, and 4.64 Å, respectively. On the other hand, compound **3** shows three interactions: one hydrogen bond with GlnB135 at a distance of 2.35 Å, and two hydrophobic bonds with ProB128 and LysB127 at distances of 5.3 Å and 4.94 Å, respectively (Table 4). Therefore, **EA** has four interactions: one hydrogen bond with AsnA110 at a distance of 2.12 Å and three hydrophobic bonds with LysA102, GlyA114, and TyrA118 at distances of 4.96 Å, 3.69 Å, and 3.19 Å, respectively (Table 4). Based on the comparison between **EA** and the synthesised compounds **2** and **3**, we found that compound **2** has more interactions than **EA**, which enhances its inhibitory activity.

**Figure 7:**
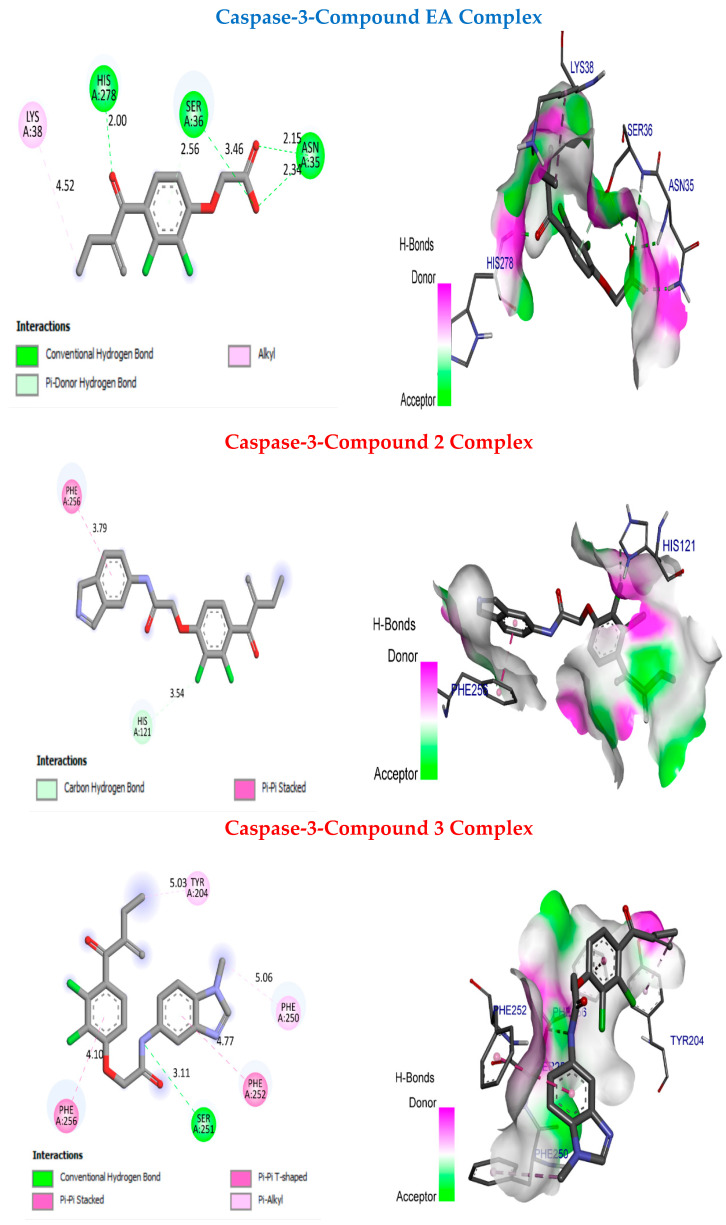
Molecular docking between the molecules **EA**, **2**, and **3** and the caspase-3 pocket.

Figure 7 presents the molecular docking for **EA**, **2**, and **3** in the human glutathione S-transferase P1-1 and caspase-3 pocket. The binding affinity values for **2** and **3** are −7.4 kcal/mol and −7.3 kcal/mol, respectively (Table 5 and Table 6). However, the binding affinity value for **EA** is −5.7 kcal/mol. It can be observed that **EA** is less stable in the caspase-3 pocket compared to the synthesised compounds **2** and **3**. Additionally, the reference compound exhibits four interactions: three hydrogen bonds with His A278, SerA36, and AsnA35 at distances of **2** Å, 2.56 Å, and 2.34 Å, respectively, and one hydrophobic bond with LysA38 at a distance of 4.52 Å. Compound **2** has two interactions, while compound **3** demonstrates five interactions: with SerA251, PheA256, PheA252, TyrA204, and PheA250 at distances of 3.11 A°, 4.77 Å, 4.10, 5.03 Å, and 5.06 Å. Notably, compound **3** displays more interactions than **EA**, which enhances its inhibitory activity. Based on these results, compounds **2** and **3** exhibit significant binding modes with human glutathione S-transferase P1-1 and caspase-3, confirming their potent inhibitory activities. By comparing the binding affinities of the complexes obtained, we found that the binding affinity of (the **2**-2GSS; **3**-2GSS) and (the **2**-4QU8; **3**-4QU8) complexes is higher than that of the (**EA**-2GSS) and (**EA**-4QU8) complexes, this comparison confirms that ligands **2** and **3** are more localised and targeting than **EA**.

## 3. Materials and Methods

### 3.1. General Procedures

All chemicals and solvents used were of analytical quality and were obtained from commercial suppliers. They were utilised without additional purification. Solvents mentioned as dry were purified with a dry station GT S100 (GlassTechnology, Geneva, Switzerland) immediately prior to use. The reactions were monitored by thin-layer chromatography (TLC) conducted on aluminium sheets coated with Merck 60 F254 silica gel (Merck, Belmopan, Belize) (thickness 0.2 mm). Compounds were visualised under ultraviolet (UV) irradiation at either 254 nm or 365 nm. Column chromatography was performed on silica gel 60 (230–400 mesh, 0.040–0.063 mm). Melting points (M.p. [°C]) were determined using samples in open capillary tubes and are reported without correction. Infrared spectra were recorded on a Thermo Scientific Nicolet iS10 (Thermo Scientific, Waltham, MA, USA) and are presented in cm^−1^. NMR spectra were acquired on a Bruker AC 250, 300, 400 MHz instrument at room temperature, using chloroform-d, methanol-d4, or acetone-d6 as the solvent. Chemical shifts (δ) are expressed in parts per million (ppm). High-resolution mass spectra (HRMS) were obtained on a Maxis Bruker 4G (Bruker, Billerica, MA, USA).

### 3.2. Optical Spectroscopy

Absorption spectra were captured using a UV-1800 Shimadzu spectrophotometer (Shimadzu, Kyoto, Japan), while fluorescence measurements were conducted with a Horiba Scientific Fluoromax-4 spectrofluorometer (Horiba Scientific, Piscataway, NJ, USA). Prior to analysis, sample solutions underwent complete degassing through argon purging. Quantum Yield Measurements were conducted to determine the efficiency of photon absorption and emission. The quantum yield (ϕ) of compounds 22 and 24 was determined using an established method relative to coumarin 153 (λex = 421 nm, λem = 531 nm in ethanol, ϕ = 0.38 in ethanol) as a reference [22]. The quantum yield in organic solvents was calculated using the following equation: ϕsample = ϕstandard · (Isample/Istandard) · (Astandard/Asample) · (nsample/nstandard)^2^, where ϕ represents the quantum yield, I denotes the area under the fluorescence band, A indicates the absorbance (within the range of 0.01–0.1 absorbance unit), and n signifies the refractive index of the solvent at 25 °C. Both compound solutions were freshly prepared prior to each spectroscopic analysis, degassed with argon for 30–40 min, and shielded from direct light throughout the procedure. Fluorescence quantum yields (ϕ) were determined in DCM at room temperature, with λex and λem (maximum emission wavelength) selected as the excitation and emission wavelengths, respectively.

### 3.3. Synthesis and Characterisation

#### 3.3.1. Preparation of Intermediate compounds

The synthesis of the key intermediate compounds **13**(**a**–**c**), **14b**, **15b**, **19**, **20**, and **21** were prepared according to references [19,21,30,31,32].

#### 3.3.2. General Experimental Procedure for the Synthesis of Products (**1**–**10**), **16**(**a**–**c**), **17b**, **18**, and **23**

To a well-stirred solution of DCC (0.62 g, 0.39 mmol), HOBt (0.60 g, 0.39 mmol), and ethacrynic acid (0.10 g, 0.33 mmol) in dry DMF (5 mL), amine (0.33 mmol) was added at 0 °C. The solution was stirred at room temperature overnight. The reaction was monitored by TLC until all the amine was consumed. The mixture was diluted with ethyl acetate (100 mL) and extracted with water (2 × 50 mL) and brine (3 × 50 mL), dried over anhydrous MgSO_4_, and concentrated under reduced pressure. The obtained residue was purified by silica gel column chromatography.

2-(2,3-dichloro-4-(2-methylenebutanoyl)phenoxy)-*N*-(1*H*-indazol-5-yl)acetamide (**1**). Following general procedure, the crude residue was purified by silica gel column chromatography, using DCM/EtOAc, 4:1 (*v*/*v*) as eluent, to obtain the expected product **1** as a white solid with a yield of 50%. M.p. 173–174 °C. ^1^H NMR (400 MHz, CDCl_3_), δ (ppm): 13.01 (s, 1H), 10.19 (s, 1H), 8.11 (s, 1H), 8.03 (s, 1H), 7.51 (d, ^3^*J_HH_* = 8.9 Hz, 1H), 7.45 (d, ^3^*J_HH_* = 8.9 Hz, 1H), 7.36 (d, ^3^*J_HH_* = 8.6 Hz, 1H), 7.20 (d, ^3^*J_HH_* = 8.6 Hz, 1H), 6.08 (s, 1H), 5.59 (s, 1H), 4.98 (s, 2H), 2.38 (q, ^3^*J_HH_* = 7.4Hz, 2H), 1.08 (t, ^3^*J_HH_* = 7.4 Hz, 3H). ^13^C NMR (101 MHz, CDCl_3_); δ (ppm): 195.6, 165.6, 156.1, 149.8, 137.5, 133.9, 132.9, 131.7, 130.0, 129.8, 128.0, 123.1, 121.6, 120.8, 112.4, 110.7, 110.6, 68.4, 23.4, 12.8. IR (neat): *ν* = 3411 (NH), 3387 (NH), 3321 (NH), 1664 (C=O Ketone), 1652 (C=O Amide) cm^−1^. HRMS (+ESI) *m*/*z*: [M + H]^+^ calculated for C_20_H_18_Cl_2_N_3_O_3_: 418.0719, found, 418.0717.

2-(2,3-dichloro-4-(2-methylenebutanoyl)phenoxy)-*N*-(1*H*-indol-5-yl)acetamide (**2**). Following general procedure, the crude residue was purified by silica gel column chromatography, using DCM/EtOAc, 4:1 (*v*/*v*) as eluent, to obtain the expected product **2** as a white solid with a yield of 62%. M.p. 159–160 °C. ^1^H NMR (400 MHz, CDCl_3_), δ (ppm): 8.57 (s, 1H), 8.36 (s, 1H), 7.97 (s, 1H), 7.41–7.29 (m, 2H), 7.27–7.18 (m, 2H), 6.92 (d, ^3^*J_HH_* = 8.5 Hz, 1H), 6.56 (s, 1H), 5.99 (s, 1H), 5.63 (s, 1H), 4.72 (s, 2H), 2.51 (q, ^3^*J_HH_* = 7.4 Hz, 2H), 1.18 (t, ^3^*J_HH_* = 7.4 Hz, 3H). ^13^C NMR (101 MHz, CDCl_3_); δ (ppm): 195.5, 164.5, 154.4, 150.2, 134.3, 133.5, 131.5, 129.2, 128.8, 128.0, 127.2, 125.4, 123.0, 116.0, 112.6, 111.3, 111.1, 102.8, 68.4, 23.4, 12.4. IR (neat): *ν* = 3383 (NH), 3313 (NH), 1663 (C=O Ketone), 1650 (C=O Amide) cm^−1^. HRMS (+ESI) *m*/*z*: [M + H]^+^ calculated for C_21_H_19_Cl_2_N_2_O_3_: 417.0767, found, 417.0765.

2-(2,3-dichloro-4-(2-methylenebutanoyl)phenoxy)-*N*-(1-methyl-1*H*-benzo[*d*]imidazol-5-yl)acetamide (**3**). Following general procedure, the crude residue was purified by silica gel column chromatography, using DCM/EtOAc, 3:1 (*v*/*v*) as eluent, to obtain the expected product **3** as a white solid with a yield of 79%. M.p. 184–185 °C. ^1^H NMR (400 MHz, CDCl_3_), δ (ppm): 8.66 (s, 1H), 8.05 (d, ^3^*J_HH_* = 1.8 Hz, 1H), 7.98 (s, 1H), 7.63 (dd, ^3^*J_HH_* = 1.8, 8.6 Hz, 1H), 7.40 (d, ^3^*J_HH_* = 8.6 Hz, 1H), 7.25 (d, ^3^*J_HH_* = 8.5 Hz, 1H), 6.97 (d, ^3^*J_HH_* = 8.5 Hz, 1H), 5.99 (s, 1H), 5.63 (s, 1H), 4.76 (s, 2H), 3.88 (s, 3H), 2.51 (q, ^3^*J_HH_* = 7.4Hz, 2H), 1.18 (t, ^3^*J_HH_* = 7.4 Hz, 3H). ^13^C NMR (101 MHz, CDCl_3_); δ (ppm): 195.4, 164.5, 154.3, 150.1, 144.5, 143.9, 134.5, 132.2, 131.7, 131.6, 128.8, 127.3, 123.1, 116.8, 112.1, 111.2, 109.5, 68.4, 31.1, 23.4, 12.4. IR (neat): *ν* = 3323 (NH), 1670 (C=O Ketone), 1658 (C=O Amide) cm^−1^. HRMS (+ESI) *m*/*z*: [M + H]^+^ calculated for C_21_H_20_Cl_2_N_3_O_3_: 432.0876, found, 432.0873.

2-(2,3-dichloro-4-(2-methylenebutanoyl)phenoxy)-*N*-(1-methyl-1*H*-indol-5-yl)acetamide (**4**). Following general procedure, the crude residue was purified by silica gel column chromatography, using DCM/EtOAc, 4:1 (*v*/*v*) as eluent, to obtain the expected product **4** as a white solid with a yield of 70%. M.p. 163–164 °C. ^1^H NMR (400 MHz, CDCl_3_), δ (ppm): 8.56 (s, 1H), 7.95 (d, ^3^*J_HH_* = 2.0 Hz, 1H), 7.38 (dd, ^3^*J_HH_* = 2.0, 8.7 Hz, 1H), 7.32 (d, ^3^*J_HH_* = 8.7 Hz, 1H), 7.25 (d, ^3^*J_HH_* = 3.1 Hz, 1H), 7.38 (d, ^3^*J_HH_* = 3.1 Hz, 1H), 6.97 (d, ^3^*J_HH_* = 8.5 Hz, 1H), 6.53–6.48 (m, 1H), 5.99 (s, 1H), 5.64 (s, 1H), 4.75 (s, 2H), 3.82 (s, 3H), 2.51 (q, ^3^*J_HH_* = 7.4 Hz, 2H), 1.18 (t, ^3^*J_HH_* = 7.4 Hz, 3H). ^13^C NMR (101 MHz, CDCl_3_); δ (ppm): 195.5, 164.3, 154.4, 150.2, 134.5, 134.3, 131.5, 129.9, 128.9, 128.7, 128.5, 127.3, 123,0, 115.5, 112.7, 111.1, 109.4, 101.1, 68.4, 32.9, 23.4, 12.4. IR (neat): *ν* = 3250 (NH), 1660 (C=O Ketone), 1652 (C=O Amide) cm^−1^. HRMS (+ESI) *m*/*z*: [M + H]^+^ calculated for C_22_H_21_Cl_2_N_2_O_3_: 431.0923, found, 431.0924.

2-(2,3-dichloro-4-(2-methylenebutanoyl)phenoxy)-*N*-(1-methyl-1*H*-indazol-5-yl)acetamide (**5**). Following general procedure, the crude residue was purified by silica gel column chromatography, using DCM/EtOAc, 4:1 (*v*/*v*) as eluent, to obtain the expected product **5** as a white solid with a yield of 82%. M.p. 176–177 °C. ^1^H NMR (400 MHz, CDCl_3_), δ (ppm): 8.63 (s, 1H), 8.14 (d, ^3^*J_HH_* = 1.5 Hz, 1H), 8.01–7.96 (m, 1H), 7.49 (dd, ^3^*J_HH_* = 1.9, 8.9 Hz, 1H), 7.40 (d, ^3^*J_HH_* = 8.9 Hz, 1H), 7.24 (d, ^3^*J_HH_* = 8.5 Hz, 1H), 6.96 (d, ^3^*J_HH_* = 8.5 Hz, 1H), 5.99 (s, 1H), 5.63 (s, 1H), 4.74 (s, 2H), 4.09 (s, 3H), 2.50 (q, ^3^*J_HH_* = 7.4 Hz, 2H), 1.18 (t, ^3^*J_HH_* = 7.4 Hz, 3H). ^13^C NMR (101 MHz, CDCl_3_); δ (ppm): 195.4, 164.6, 154.3, 150.1, 137.7, 134.5, 132.8, 131.5, 129.8, 128.8, 127.3, 124.0, 123.0, 120.7, 112.0, 111.2, 109.4, 68.4, 35.6, 23.4, 12.4. IR (neat): *ν* = 3392 (NH), 1666 (C=O Ketone), 1655 (C=O Amide) cm^−1^. HRMS (+ESI) *m*/*z*: [M + H]^+^ calculated for C_21_H_20_Cl_2_N_3_O_3_: 432.0876, found, 432.0874.

1-(4-(2-(1*H*-indazol-1-yl)-2-oxoethoxy)-2,3-dichlorophenyl)-2-methylenebutan-1-one (**6**). Following general procedure, the crude residue was purified by silica gel column chromatography, using DCM/EtOAc, 4:1 (*v*/*v*) as eluent, to obtain the expected product **6** as a white solid with a yield of 62%. M.p. 168–179 °C. ^1^H NMR (400 MHz, CDCl_3_), δ (ppm): 8.44 (d, ^3^*J_HH_* = 8.4 Hz, 1H), 8.23 (s, 1H), 7.81 (d, ^3^*J_HH_* = 8.4 Hz, 1H), 7.64 (t, ^3^*J_HH_* = 7.6 Hz, 1H), 7.45 (t, ^3^*J_HH_* = 7.6 Hz, 1H), 7.15 (d, ^3^*J_HH_* = 8.5 Hz, 1H), 6.94 (d, ^3^*J_HH_* = 8.5 Hz, 1H), 5.96 (s, 1H), 5.71 (s, 2H), 5.65 (s, 1H), 2.49 (q, ^3^*J_HH_* = 7.4 Hz, 2H), 1.17 (t, ^3^*J_HH_* = 7.4 Hz, 3H). ^13^C NMR (101 MHz, CDCl_3_); δ (ppm): 195.8, 166.4, 155.6, 150.1, 141.2, 139.0, 133.8, 131.5, 130.1, 128.5, 126.8, 126.0, 125.2, 123.4, 121.2, 115.1, 111.0, 67.4, 23.4, 12.4. IR (neat): *ν* = 1669 (C=O Ketone), 1660 (C=O Amide) cm^−1^. HRMS (+ESI) *m*/*z*: [M + H]^+^ calculated for C_20_H_17_Cl_2_N_2_O_3_: 403.0610, found, 403.0612.

2-(2,3-dichloro-4-(2-methylenebutanoyl)phenoxy)-*N*-(2-methyl-1,3-dioxoisoindolin-5-yl)acetamide (**7**). Following general procedure, the crude residue was purified by silica gel column chromatography, using DCM/EtOAc, 4:1 (*v*/*v*) as eluent, to obtain the expected product **7** as a white solid with a yield of 47%. M.p. 178–179 °C. ^1^H NMR (400 MHz, CDCl_3_), δ (ppm): 8.91 (s, 1H), 8.16 (d, ^3^*J_HH_* = 1.9 Hz, 1H), 8.00 (dd, ^3^*J_HH_* = 1.9, 8.1 Hz, 1H), 7.88 (d, ^3^*J_HH_* = 8.1 Hz, 1H), 7.26 (d, ^3^*J_HH_* = 8.5 Hz, 1H), 6.96 (d, ^3^*J_HH_* = 8.5 Hz, 1H), 6.00 (s, 1H), 5.62 (s, 1H), 4.77 (s, 2H), 3.21 (s, 3H), 2.51 (q, ^3^*J_HH_* = 7.4 Hz, 2H), 1.18 (t, ^3^*J_HH_* = 7.4 Hz, 3H). ^13^C NMR (101 MHz, CDCl_3_); δ (ppm): 195.3, 167.8 (2C), 165.0, 153.9, 150.1, 142.0, 134.9, 134.0, 131.7, 128.8, 127.9, 127.3, 124.4, 124.0, 123.0, 114.2, 111.2, 68.2, 24.0, 23.4, 12.4. IR (neat): *ν* = 3350 (NH), 1770 (C=O Ketone), 1703 (C=O Amide), 1658 (C=O Amide) cm^−1^. HRMS (+ESI) *m*/*z*: [M + H]^+^ calculated for C_22_H_19_Cl_2_N_2_O_5_: 461.0665, found, 461.0664.

2-(2,3-dichloro-4-(2-methylenebutanoyl)phenoxy)-*N*-(pyridin-2-yl)acetamide (**8**). Following general procedure, the crude residue was purified by silica gel column chromatography, using DCM/EtOAc, 4:1 (*v*/*v*) as eluent, to obtain the expected product **8** as a white solid with a yield of 40%. M.p. 144–146 °C. ^1^H NMR (400 MHz, CDCl_3_), δ (ppm): 9.02 (br, 1H), 8.34–8.32 (m, 1H), 8.25–8.21 (m, 1H), 7.76–7.69 (m, 1H), 7.18 (d, ^3^*J_HH_* = 8.5 Hz, 1H), 7.11–7.06 (m, 1H), 6.90 (d, ^3^*J_HH_* = 8.5 Hz, 1H), 5.94 (s, 1H), 5.58 (s, 1H), 4.70 (s, 2H), 2.45 (q, ^3^*J_HH_* = 7.4 Hz, 2H), 1.13 (t, ^3^*J_HH_* = 7.4 Hz, 3H). ^13^C NMR (101 MHz, CDCl3); δ (ppm): 195.5, 165.1, 154.5, 150.3, 150.1, 148.2, 138.4, 134.5, 131.5, 130.7, 128.7, 127.1, 120.6, 114.2, 111.2, 68.4, 23.4, 12.3. IR (neat): *ν* = 3445 (NH), 1682 (C=O Ketone), 1660 (C=O Amide) cm^−1^. HRMS (+ESI) *m*/*z*: [M + H]^+^ calculated for C_18_H_17_Cl_2_N_2_O_3_: 379.0610, found, 379.0609.

2-(2,3-dichloro-4-(2-methylenebutanoyl)phenoxy)-*N*-(pyridin-3-yl)acetamide (**9**). Following general procedure, the crude residue was purified by silica gel column chromatography, using DCM/EtOAc, 4:1 (*v*/*v*) as eluent, to obtain the expected product **9** as a white solid with a yield of 53%. M.p. 145–146 °C. ^1^H NMR (400 MHz, CDCl_3_), δ (ppm): 8.72 (d, ^3^*J_HH_* = 2.5 Hz, 1H), 8.70 (br, 1H), 8.43 (dd, ^3^*J_HH_* = 1.2, 4.8 Hz, 1H), 8.22–8.19 (m, 1H), 7.33 (dd, ^3^*J_HH_* = 4.8, 8.3 Hz, 1H), 7.23 (d, ^3^*J_HH_* = 8.5 Hz, 1H), 6.95 (d, ^3^*J_HH_* = 8.5 Hz, 1H), 5.98 (s, 1H), 5.61 (s, 1H), 4.74 (s, 2H), 2.48 (q, ^3^*J_HH_* = 7.4 Hz, 2H), 1.16 (t, ^3^*J_HH_* = 7.4 Hz, 3H). ^13^C NMR (101 MHz, CDCl_3_); δ (ppm): 195.3, 165.3, 154.2, 150.0, 146.0, 141.4, 134.7, 133.5, 131.6, 128.8, 127.3, 127.2, 123.8, 122.9, 111.3, 68.0, 23.2, 12.2. IR (neat): *ν* = 3443 (NH), 1687 (C=O Ketone), 1659 (C=O Amide) cm^−1^. HRMS (+ESI) *m*/*z*: [M + H]^+^ calculated for C_18_H_17_Cl_2_N_2_O_3_: 379.0610, found, 379.0608.

2-(2,3-dichloro-4-(2-methylenebutanoyl)phenoxy)-*N*-(pyridin-4-yl)acetamide (**10**). Following general procedure, the crude residue was purified by silica gel column chromatography, using DCM/EtOAc, 4:1 (*v*/*v*) as eluent, to obtain the expected product **10** as a white solid with a yield of 51%. M.p. 145–146 °C. ^1^H NMR (400 MHz, CDCl_3_), δ (ppm): 8.73 (m, 2H), 8.63 (br, 1H), 7.62–7.60 (m, 2H), 7.25 (d, ^3^*J_HH_* = 8.5 Hz, 1H), 6.95 (d, ^3^*J_HH_* = 8.5 Hz, 1H), 6.00 (s, 1H), 5.62 (s, 1H), 4.74 (s, 2H), 2.50 (q, ^3^*J_HH_* = 7.4 Hz, 2H), 1.20 (t, ^3^*J_HH_* = 7.4 Hz, 3H). ^13^C NMR (101 MHz, CDCl_3_); δ (ppm): 195.3, 165.4, 154.0, 150.9, 150.1, 146.6, 134.9, 131.7, 131.4, 128.9, 127.3 (2C), 123.0, 111.3 (2C), 68.3, 23.3, 12.3. IR (neat): *ν* = 3336 (NH), 1682 (C=O Ketone), 1660 (C=O Amide) cm^−1^. HRMS (+ESI) *m*/*z*: [M + H]^+^ calculated for C_18_H_17_Cl_2_N_2_O_3_: 379.0610, found, 379.0608.

2-(2,3-dichloro-4-(2-methylenebutanoyl)phenoxy)*-N*-(2-(3-(p-tolyl)ureido)ethyl)acetamide (**16a**). Following general procedure, the crude residue was purified by silica gel column chromatography, using DCM/EtOAc, 3:2 (*v*/*v*) as eluent, to obtain the expected product **16a** as a white solid with a yield of 70%. M.p. 158–160 °C. ^1^H NMR (400 MHz, CD_3_OD), δ (ppm): 7.21 (d, ^3^*J_HH_* = 8.5 Hz, 2H), 7.14 (d, ^3^*J_HH_* = 9.0 Hz, 1H), 7.06–7.00 (m, 3H), 6.01 (s, 1H), 5.56 (s, 1H), 4.71 (s, 2H), 3.46–3.37 (m, 4H), 2.45 (q, ^3^*J_HH_* = 7.4 Hz, 2H), 2.27 (s, 3H), 1.15 (t, ^3^*J_HH_* = 7.4 Hz, 3H). ^13^C NMR (101 MHz, CD_3_OD); δ (ppm): 195.8, 168.8, 157.2, 155.5, 150.1, 136.7, 133.5, 131.7, 130.3, 128.8 (2C), 128.5, 127.0, 122.5, 119.2 (2C), 111.4, 68.0, 39.5, 38.8, 23.0, 19.3, 11.5. IR (neat): *ν* = 3350 (NH), 3323 (NH), 1652 (C=O Ketone), 1605 (C=O Amide) cm^−1^. HRMS (+ESI) *m*/*z*: [M + H]^+^ calculated for C_23_H_26_Cl_2_N_3_O_4_: 478.1294, found: 478.1291.

2-(2,3-dichloro-4-(2-methylenebutanoyl)phenoxy)-*N*-(2-(3-(4 fluorophenyl)ureido)ethyl) acetamide (**16b**). Following general procedure, the crude residue was purified by silica gel column chromatography, using DCM/EtOAc, 3:2 (*v*/*v*) as eluent, to obtain the expected product **16b** as a white solid with a yield of 66%. M.p. 160–161 °C. ^1^ NMR (400 MHz, CD_3_COCD_3_), δ (ppm): 7.99 (br, 1H), 7.63 (br, 1H), 7.45–7.41(m, 2H), 7.23 (d, ^3^*J_HH_* = 9.0 Hz, 1H), 7.13 (d, ^3^*J_HH_* = 9.0 Hz, 1H), 6.94–6.90 (m, 2H), 5.97 (s, 1H), 5.88 (br, 1H), 5.54 (s, 1H), 4.64 (s, 2H), 3.38–3.35 (m, 4H), 2.46 (q, ^3^*J_HH_* = 7.4 Hz, 2H), 1.08 (t, ^3^*J_HH_* = 7.4 Hz, 3H). ^13^C NMR (101 MHz, CD_3_COCD_3_); δ (ppm): 194.7, 167.2, 159.4, 155.5, 155.7, 150.1 (2C), 136.9, 133.7 (2C), 128.1, 127.4, 119.8, 119.7, 114.9, 114.7, 111.8, 68.5, 39.8, 39.4, 23.2, 11.8. IR (neat): *ν* = 3339 (NH), 3337 (NH), 1650 (C=O Ketone), 1601 (C=O Amide) cm^−1^. HRMS (+ESI) *m*/*z*: [M + H]^+^ calculated for C_22_H_23_Cl_2_FN_3_O_4_: 482.1044, found: 482.1040.

2-(2,3-dichloro-4-(2-methylenebutanoyl)phenoxy)-*N*-(2-(3-(4-methoxyphenyl)ureido)ethyl)acetamide (**16c**). Following general procedure, the crude residue was purified by silica gel column chromatography, using DCM/EtOAc, 3:2 (*v*/*v*) as eluent, to obtain the expected product **16c** as a white solid with a yield of 75%. M.p. 166–167 °C. ^1^NMR (400 MHz, CD_3_COCD_3_), δ (ppm): 7.86 (br, 1H), 7.74 (br, 1H), 7.37 (d, ^3^*J_HH_* = 8.5 Hz, 2H), 7.27 (d, ^3^*J_HH_* = 9.0 Hz, 1H), 7.18 (d, ^3^*J_HH_* = 9.0 Hz, 1H), 6.80 (d, ^3^*J_HH_* = 8.5 Hz, 2H), 6.02 (br, 1H), 5.91 (s, 1H), 5.59 (s, 1H), 4.72 (s, 2H), 3.74 (s, 3H), 3.44–3.37 (m, 4H), 2.44 (q, ^3^*J_HH_* = 7.4 Hz, 2H), 1.13 (t, ^3^*J_HH_* = 7.4 Hz, 3H). ^13^C NMR (101 MHz, CD_3_COCD_3_); δ (ppm): 194.8, 167.0, 155.5, 154.8, 150.1, 133.6, 133.5, 130.1, 128.2, 127.3, 122.2, 120.0 (2C), 119.9, 113.7 (2C), 111.8, 68.4, 54.7, 39.6, 39.4, 23.1, 11.9. IR (neat): *ν* = 3300 (NH), 3315 (NH), 1647 (C=O Ketone), 1610 (C=O Amide) cm^−1^. HRMS (+ESI) *m*/*z*: [M + H]^+^ calculated for C_23_H_26_Cl_2_N_3_O_5_: 494.1244, found: 494.1242.

2-(2,3-dichloro-4-(2-methylenebutanoyl)phenoxy)-*N*-(2-(3-(4-fluorophenyl)thioureido)ethyl) acetamide (**17b**). Following general procedure, the crude residue was purified by silica gel column chromatography, using DCM/EtOAc, 3:2 (*v*/*v*) as eluent, to obtain the expected product **17b** as a white solid with a yield of 68%. M.p. 170–173 °C. ^1^ NMR (400 MHz, CDCl_3_), δ (ppm): 7.26–7.23 (m, 3H), 7.20–7.18 (m, 2H), 7.19 (d, ^3^*J_HH_* = 9.0 Hz, 2H), 6.60 (br, 1H), 5.98 (s, 1H), 5.61 (s, 1H), 4.54 (s, 2H), 3.89–3.86 (m, 2H), 3.63–3.59 (m, 2H), 2.48 (q, ^3^*J_HH_* = 7.4 Hz, 2H), 1.17 (t, ^3^*J_HH_* = 7.4 Hz, 3H). ^13^C NMR (101 MHz, CDCl_3_); δ (ppm): 195.5, 181.8, 168.1, 162.7, 160.3, 154.4, 150.1, 134.3, 131.5, 128.8, 127.9, 127.8, 127.1, 123.0, 117.0, 116.8, 110.9, 68.1, 45.4, 39.0, 23.3, 12.3. IR (neat): *ν* = 3336 (NH), 3333 (NH), 1655 (C=O Ketone), 1610 (C=S Thiourea) cm^−1^. HRMS (+ESI) *m*/*z*: [M + H]^+^ calculated for C_22_H_23_Cl_2_FN_3_O_3_S: 498.0640, found: 498.0632.

4-(2-(2,3-dichloro-4-(2-methylenebutanoyl)phenoxy)acetyl)-*N*-(4-fluorophenyl)piperazine-1-carboxamide (**18**). Following general procedure, the crude residue was purified by silica gel column chromatography, using DCM/EtOAc, 3:2 (*v*/*v*) as eluent, to obtain the expected product **18** as a white solid with a yield of 61%. M.p. 179–181 °C. ^1^NMR (400 MHz, CDCl_3_), δ (ppm): 7.32–7.27 (m, 2H), 7.17 (d, ^3^*J_HH_* = 9.0 Hz, 1H), 7.03–6.98 (m, 3H), 6.58 (br, 1H), 5.98 (s, 1H), 5.62 (s, 1H), 4.87 (s, 2H), 3.71–3.68 (m, 4H), 3.60–3.49 (m, 4H), 2.48 (q, ^3^*J_HH_* = 7.4 Hz, 2H), 1.16 (t, ^3^*J_HH_* = 7.4 Hz, 3H). ^13^C NMR (101 MHz, CDCl_3_); δ (ppm): 195.8, 165.4, 160.3, 157.9, 154.9, 150.1, 134.4, 133.9, 131.4, 128.9, 127.0, 122.7, 122.3, 122.2, 115.6, 115.4, 110.6, 68.6, 45.2, 43.9, 43.8, 41.8, 23.4, 12.3. IR (neat): *ν* = 3342 (NH), 3333 (NH), 1661 (C=O Ketone), 1605 (C=O Urea) cm^−1^. HRMS (+ESI) *m*/*z*: [M + H]^+^ calculated for C_24_H_25_Cl_2_FN_3_O_4_: 508,1020, found: 508,1017.

2-(2,3-dichloro-4-(2-methylenebutanoyl)phenoxy)-*N*-(3-(8,9-dihydro-7*H*-5l2-pyrazolo[1′,2′:2,3] [1,2,3]triazolo[4,5-b]pyrazin-8-yl)prop-2-yn-1-yl)acetamide (**22**). In a dry sealed tube under argon, the pharmacophore **19** (5 mmol) is solubilised in an acetonitrile/DMF (4/1: *v*/*v*); then distilled triethylamine (10 mmol) and alkyne compound **21** (10 mmol) are added. The reaction mixture is degassed with argon for 5 to 10 min; then Pd(Ph_3_)_2_Cl_2_ (10 mol%) and CuI (10 mol%) are added. The crude residue was purified by silica gel column chromatography, using DCM/EtOAc, 3:1 (*v*/*v*) as eluent, to obtain the expected product **22** as a white solid with a yield of 51%. M.p. 160 °C (degradation). ^1^NMR (400 MHz, CD_3_COCD_3_), δ (ppm): 8.60 (s, 1H), 8.43 (d, ^3^*J_HH_* = 2.6 Hz, 1H), 8.29 (s, 1H), 7.96 (d, ^3^*J_HH_* = 2.6 Hz, 1H), 7.92 (br, 1H), 7.36 (d, ^3^*J_HH_* = 9.0 Hz, 1H), 7.23 (d, ^3^*J_HH_* = 8.6 Hz, 1H), 6.03 (s, 1H), 4.55 (s, 1H), 4.83 (s, 2H), 4.42 (d, ^3^*J_HH_* = 5.9 Hz, 2H), 2.43 (q, ^3^*J_HH_* = 7.4Hz, 2H), 1.11 (t,^3^*J_HH_* = 7.4 Hz, 3H). ^13^C NMR (101 MHz, CD_3_COCD_3_); δ (ppm): 194.8, 171.1, 166.6, 155.4, 150.2, 143.2, 133.7, 130.1, 128.2, 127.4, 112.9, 111.9, 110.7, 110.4, 107.2, 107.0, 106.6, 105.1, 89.2, 71.4, 68.3, 23.1, 11.9. IR (neat): *ν* = 3421 (NH), 2115 (C≡C), 1667 (C=O Ketone), 1605 (C=O Amide) cm^−1^. HRMS (+ESI) *m*/*z*: [M + H]^+^ calculated for C_23_H_19_Cl_2_N_6_O_3_: 497.0890, found: 497.0887.

2-(2,3-dichloro-4-(2-methylenebutanoyl)phenoxy)-*N*-(2-hydroxyethyl)acetamide (**23**). Following general procedure, the crude residue was purified by silica gel column chromatography, using DCM/EtOAc, 1:1 (*v*/*v*) as eluent, to obtain the expected product **23** as a white solid with a yield of 62%. M.p. 152–150 °C. ^1^H NMR (400 MHz, CDCl_3_), δ (ppm): 7.22–7.20 (m, 2H), 6.89 (d, ^3^*J_HH_* = 8.5 Hz, 1H), 5.98 (s, 1H), 5.61 (s, 1H), 4.62 (s, 2H), 3.87–3.79 (m, 2H), 3.60 (q, ^3^*J_HH_* = 5.5 Hz, 2H), 2.50 (q, ^3^*J_HH_* = 7.4 Hz, 2H), 1.17 (t, ^3^*J_HH_* = 7.4 Hz, 3H). ^13^C NMR (101 MHz, CDCl_3_); δ (ppm): 195.5, 167.7, 154.4, 150.2, 134.2, 131.5, 128.7, 127.2, 123.0, 110.9, 68.2, 62.0, 41.9, 23.4, 12.3. IR (neat): *ν* = 3405 (OH), 1668 (C=O Ketone), 1662 (C=O Amide) cm^−1^. HRMS (+ESI) *m*/*z*: [M + H]^+^ calculated for C_15_H_18_Cl_2_NO_4_: 346.0607, found, 346.0607.

8-((2-(2-(2,3-dichloro-4-(2-methylenebutanoyl)phenoxy) acetamido)ethoxy)carbonyl) pyrazolo [1′,2′:1,2][1,2,3]triazolo[4,5-b]pyrazin-6-ium-5-ide (**24**). Acid **20** (0.59 g, 0.289 mmol), pentafluorophenol (0.58 g, 0.318 mmol), and EDC (0.58 g, 0.318 mmol) are mixed in DMF (5 mL) at 0 °C. The reaction mixture is stirred at 0 °C for one hour; then one equivalent of compound **23** (0.10 g, 0.289 mmol) is added. After 16 h, the mixture is extracted with ethyl acetate and the combined organic phases are washed with saturated aqueous sodium chloride, dried over magnesium sulphate, and concentrated under pressure. The crude residue is purified by silica gel column chromatography using DCM/EtOAc, 3:1 (*v*/*v*) as eluent, to obtain the expected product **24** as a white solid with a yield of 56%. M.p. 172 °C (degradation). ^1^H NMR (400 MHz, CDCl_3_), δ (ppm): 8.59–8.50 (m, 2H), 8.25 (s, 1H), 8.01 (d, ^3^*J_HH_* = 2.1 Hz, 1H), 7.22–7.19 (m, 2H), 6.88 (d, ^3^*J_HH_* = 8.5 Hz, 1H), 5.94 (s, 1H), 5.56 (s, 1H), 4.63 (s, 2H), 4.56 (q, ^3^*J_HH_* = 5.3 Hz, 2H), 3.85 (q, ^3^*J_HH_* = 5.6 Hz, 2H), 2.45 (q, ^3^*J_HH_* = 7.4Hz, 2H), 1.14 (t, ^3^*J_HH_* = 7.4 Hz, 3H). ^13^C NMR (101 MHz, CDCl_3_); δ (ppm): 195.3, 167.2, 160.9, 154.3, 152.6, 150.1, 144.3, 134.3, 131.5, 131.3, 128.8, 128.7, 127.2, 122.7, 117.4, 111.9, 110.9, 108.9, 68.2, 64.1, 38.3, 23.3, 12.3. IR (neat): *ν* = 3432 (NH), 1667 (C=O Ketone), 1652 (C=O Amide), cm^−1^. HRMS (+ESI) *m*/*z*: [M + H]^+^ calculated for C_23_H_21_Cl_2_N_6_O_5_: 531.0945, found, 531.0943.

### 3.4. Biological Evaluation

#### Cell Culture and Proliferation Assay

Cancer cell lines were sourced from the American Type Culture Collection (Rockville, MD, USA) or the European Collection of Cell Culture (ECACC, Salisbury, UK) and were cultured following the provided instructions. The human HCT-116 colorectal carcinoma cells were cultured in Gibco McCoy’s 5A with 10% fetal calf serum (FCS) and 1% glutamine, while HL60 myelogenous leukaemia cells were cultivated in RPMI 1640 supplemented with 10% FCS and 1% glutamine. The cells were maintained at 37 °C in a humidified atmosphere with 5% CO_2_. Cell growth inhibition was assessed using an MTS assay per the manufacturer’s guidelines (Promega, Madison, WI, USA). In brief, cells were seeded in 96-well plates (2.5 × 10^3^ cells/well) with 100 μL of growth medium. After 24 h, the cells were exposed to the test compounds at ten different concentrations. Following 72 h of incubation, 20 μL of Cell Titer 96^®^ AQueous One Solution Reagent (Promega Corporation, Madison, WI, USA) was added and incubated for 2 h before recording absorbance at 490 nm using a spectrophotometric plate reader (Promega Corporation, Madison, WI, USA). Dose–response curves were generated using Graph Prism software (version 9.5.1) (https://www.graphpad.com/demos/, accessed on 11 February 2024), and IC_50_ values were calculated using polynomial curves (four- or five-parameter logistic equations) with the same software.

### 3.5. Molecular Modelling

#### 3.5.1. Drug-Likeness

Drug-likeness studies are a crucial step in the drug discovery and development process and are an ongoing area of research and development in the field of medicinal chemistry. Drug-likeness parameters were calculated in silico using the SwissADME web server [27]. This allowed us to identify molecules that meet the optimal requirements for drug-like molecules, based on synthetic accessibility, the Lipinski rule [24], the Egan rule [26], and the Veber rule [25].

#### 3.5.2. Molecular Docking

In this study, the objective of molecular docking was to predict and analyse the binding and interactions of selected molecules identified through drug-likeness studies with two proteins, Human glutathione s-transferase (pdb:2GSS) and caspase-3 (pdb:4QU8), obtained from the RCSB Protein Data Bank [33]. Autodock Vina software [34] was used for molecular docking simulations, and the resulting data were analysed using Discovery Studio Client [35]. The ligand and protein were prepared using Autodock Vina, including steps such as removing water molecules, adding Kollman charges, computing Gasteiger charges, adding hydrogen atoms (only polar), and saving the protein as pdbqt. Grid-based docking was performed, using a three-dimensional grid with specific coordinates and dimensions for the active site of the 2GSS protein (X = 19.614 Å, Y = 12.147 Å, Z = 17.401 Å, dimensions 60 × 60 × 60 Å^3^) and for caspase-3 (X = 6.728 Å, Y = 107.154 Å, Z = 107.11 Å, dimensions 40 × 40 × 40 Å^3^).

## 4. Conclusions

In conclusion, novel **EA** analogues containing nitrogen heterocyclic, urea, and thiourea moieties have been synthesised by modifying the carboxylic acid part. In addition, two **PyTAP**-based fluorescent **EA** analogues were prepared. All the synthesised compounds were evaluated and screened for their in vitro anti-proliferative activities against two cancer cell lines (HL60 and HCT116). The obtained results show that selected compounds (**1**–**3**, **10**, **16**(**a**–**b**), and **24**)) displayed excellent anti-proliferative activity against HL-60 cells, with IC50 ranging between 0.86 and 2.37 μM. Compounds **2** and **10** were very promising with cell viabilities of 28% and 48% for the HCT116 treated cells, respectively. We also noticed that compounds bearing the urea moiety (**16a** and **16b**) exhibited almost similar anti-proliferative activity against the HL60 cell line as those containing the aminoheterocyclic groups, with IC50 values of 2.37 μM and 0.86 μM, respectively. Preliminary fluorescence assays showed good cell penetration and distribution of fluorescent **EA** analogues at 10 μM on HCT116 cells. Drug-likeness properties were also performed, indicating that most of the **EA** analogues exhibit a high absorption. Based on the anti-proliferative activities and drug-likeness property results, molecular docking analysis was accomplished for three molecules against two human proteins, glutathione S-transferase P1-1 pocket and caspase-3, as target enzymes. The results of this study showed that compounds **2** and **3** exhibited prominent binding modes, such as hydrogen bond interactions and hydrophobic interactions with the target enzymes. These interesting results will help us in the future with the development of new anticancer agents based on **EA** analogues that are capable of inhibiting the proliferation of cancer cells.

## Figures and Tables

**Table 1:** Spectroscopic characterisation of the compounds **22** and **24** in DCM.

Compounds	22	24
λ_ex_ ^a^ (nm)	416	380
λ_em_ ^b^ (nm)	481	470
ε_max_ ^c^	8347	2678
δ ^d^	63	128
ϕ ^e^	0.43	0.93
Brightness	3584	2513
Colour	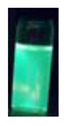	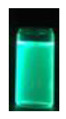

^a^ Apparent maxima of absorption bands. ^b^ Apparent maxima of emission band. ^c^ Units: L·mol^−1^·cm^−1^. ^d^ Stokes shift (nm). ^e^ ϕ is the relative fluorescence quantum yield estimated by using coumarin 153 (ϕ = 0.38 in ethanol) as a reference standard [22].

**Table 2:** IC_50_ values of selected **EA** analogues against HL60 (µM).

Compounds	1	2	3	10	16a	16b	16c	17b	24
IC_50_ HL60 (µM)	1.82	1.53	1.05	1.64	2.37	0.86	3.36	6.43	1.33

**Table 3:** The results of physicochemical properties of the synthetised compounds.

Compounds	Log P	HBD	HBA	NROTB	S. Has	MW	ABS	TPSA	Lipinski’s Violations
	≤5	<5	<10	-	1 < SA < 10	<500	-	-	
**EA**	2.55	1	4	6	2.43	303.14	High	63.6	0
**1**	2.71	2	4	8	2.81	418.27	High	84.08	0
**2**	2.93	2	3	8	2.8	417.29	High	71.19	0
**3**	2.79	1	4	8	2.88	432.30	High	73.22	0
**4**	3.14	1	3	8	2.89	431.31	High	60.33	0
**5**	2.93	1	4	8	2.9	432.3	High	73.22	0
**6**	3.66	0	4	7	2.92	403.26	High	61.19	0
**7**	2.48	1	5	8	3.00	461.29	High	92.78	0
**8**	2.56	1	4	8	2.70	379.24	High	68.29	0
**9**	2.15	1	4	8	2.70	379.24	High	68.29	0
**10**	2.15	1	4	8	2.61	379.24	High	68.29	0
**16a**	3.15	3	4	13	3.34	478.37	High	96.53	0
**16b**	3.32	3	5	13	3.22	482.33	High	96.53	0
**16c**	2.63	3	5	14	3.93	494.37	High	105.76	0
**17b**	3.30	3	4	13	3.26	498.4	High	111.55	0
**18**	3.33	1	5	10	3.38	508.37	High	78.95	1

Log P: Consensus of calculated lipophilicity; HBD: Num. H-bond donors; HBA: Num. H-bond acceptors; NROTB: Num rotatable bonds; S. Has: Synthetic accessibility; MW: Molecular weight; ABS: Absorption; TPSA: Topological polar surface area.

**Table 4:** Drug-likeness properties of the synthetised compounds.

Compounds	Lipinski	Veber	Egan	Bioavailability Score	Lipinski’s Violations
**EA**	Yes	Yes	Yes	0.55	0
**1**	Yes	Yes	Yes	0.55	0
**2**	Yes	Yes	Yes	0.55	0
**3**	Yes	Yes	Yes	0.55	0
**4**	Yes	Yes	Yes	0.55	0
**5**	Yes	Yes	Yes	0.55	0
**6**	Yes	Yes	Yes	0.55	0
**7**	Yes	Yes	Yes	0.55	0
**8**	Yes	Yes	Yes	0.55	0
**9**	Yes	Yes	Yes	0.55	0
**10**	Yes	Yes	Yes	0.55	0
**16a**	Yes	No	Yes	0.55	0
**16b**	Yes	No	Yes	0.55	0
**16c**	Yes	No	Yes	0.55	0
**17b**	Yes	No	Yes	0.55	0
**18**	Yes	No	Yes	0.55	1

**Table 5:** The resume of the results of compounds **EA, 2**, and **3** in the caspase-3 pocket.

Compounds	Caspase-3		
**EA**	Hydrogen bond interactions	Hydrophobic interactions	Bond energies (kcal\mol)
	Conventional H-bond: His A278, Ser A36, Asn A35	Alkyl and π-alkyl: Lys A38	−5.7
**2**	Carbon hydrogen bond: His A121	π–π stacked: Phe A256	−7.4
**3**	Conventional H-bond: Ser A251	Alkyl and π-alkyl: Phe A250, Tyr A204 π–π stacked: Phe A256, Phe A252	−7.3

**Table 6:** The resume of the results of compounds **EA, 2,** and **3** in the Human glutathione S-transferase P1-1 pocket.

Compounds	Human Glutathione S-Transferase P1-1		
**EA**	Hydrogen bond interactions	Hydrophobic interactions	Bond energies (kcal\mol)
	Conventional H-bond: Asn A110	π-sigma: Tyr A118 π-alkyl: Lys A102 Halogen: Gly A114	−6.2
**2**	Carbon hydrogen bond: Ile A143 Conventional H-bond: Gln A135	π-alkyl: Val A144, Leu B48 π–π stacked: Phe B8 π sigma: LeuA88	−6.7
**3**	Conventional H-bond: Gln A135	π-alkyl: Pro B128, Lys B127	−7.00

## Data Availability

All data supporting this research are available Supplementary Materials.

## Supplementary Materials

The following supporting information can be downloaded at: https://www.mdpi.com/article/10.3390/molecules29071437/s1, Spectral Data of All the Synthesised Products, NMR Spectra of All the Products.
