# Supplementary material for: Design, Synthesis, Computational Studies, and Anti-Proliferative Evaluation of Novel Ethacrynic Acid Derivatives Containing Nitrogen Heterocycle, Urea, and Thiourea Moieties as Anticancer Agents
Source: Molecules. 2024 Mar 23;29(7):1437. doi: 10.3390/molecules29071437 (PMC11013014; doi:10.3390/molecules29071437)
Supplement: Supplementary file 1 [file molecules-29-01437-s001.zip › molecules-2892834-supplementary.pdf]

SUPPORTING INFORMATION

Design, Synthesis, Computational Studies, and Anti-proliferative Evaluation of Novel Ethacrynic Acid Derivatives Containing Nitrogen Heterocycle, Urea, and Thiourea Moieties as Anticancer Agents

Abdelmoula El Abbouchi[1,2], Khaoula Mkhayar[3], Souad Elkhattabi[3], Nabil El Brahmi[1], Marie-Aude Hiebel[2], Jérôme Bignon[4], Gérald Guillaumet[1,2,*], Franck Suzenet[2,*] and Saïd El Kazzouli[1,*]

[1] Euromed Research Center, Euromed Faculty of Pharmacy and School of Engineering in Biomedical and Biotechnology, Euromed University of Fes (UEMF), Meknes Road, Fez 30000, Morocco

[2] Institut de Chimie Organique et Analytique, Université d'Orléans, UMR CNRS 7311, BP 6759, CEDEX 2, 45067 Orléans, France

[3] Laboratory of Engineering, Systems and Applications, National School of Applied Sciences, Sidi Mohamed Ben Abdellah-Fez University, BP Box 72, Fez, Morocco

[4] Institut de Chimie des Substances Naturelles, CNRS, Université Paris-Saclay, 91190 Gif-sur-Yvette, France

* Correspondence: gerald.guillaumet@univ-orleans.fr (G.G.); franck.suzenet@univ-orleans.fr (F.S.);
s.elkazzouli@ueuromed.org (S.E.K.)

Contents :

NMR (^{1}H and ^{13}C) for compounds **1-10, 16-18, 22, 23** and **24** **2-19**

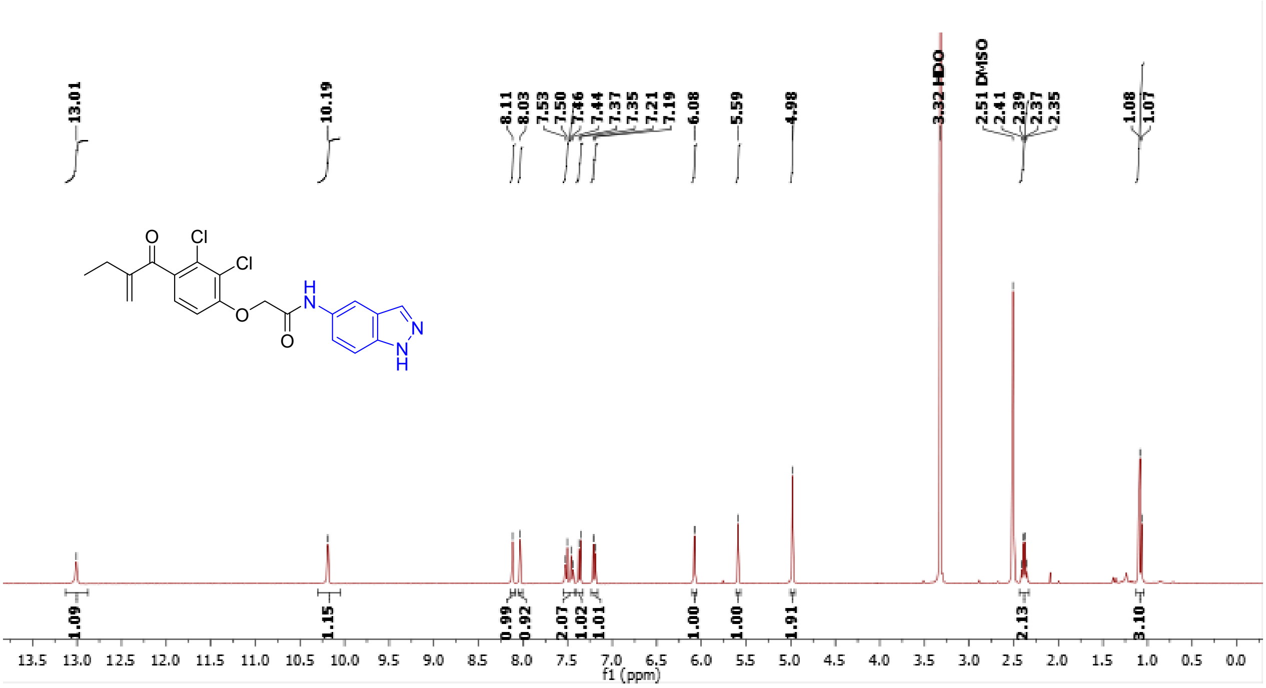

^{1}H NMR spectrum (400 MHz, DMSO) of compound **1**

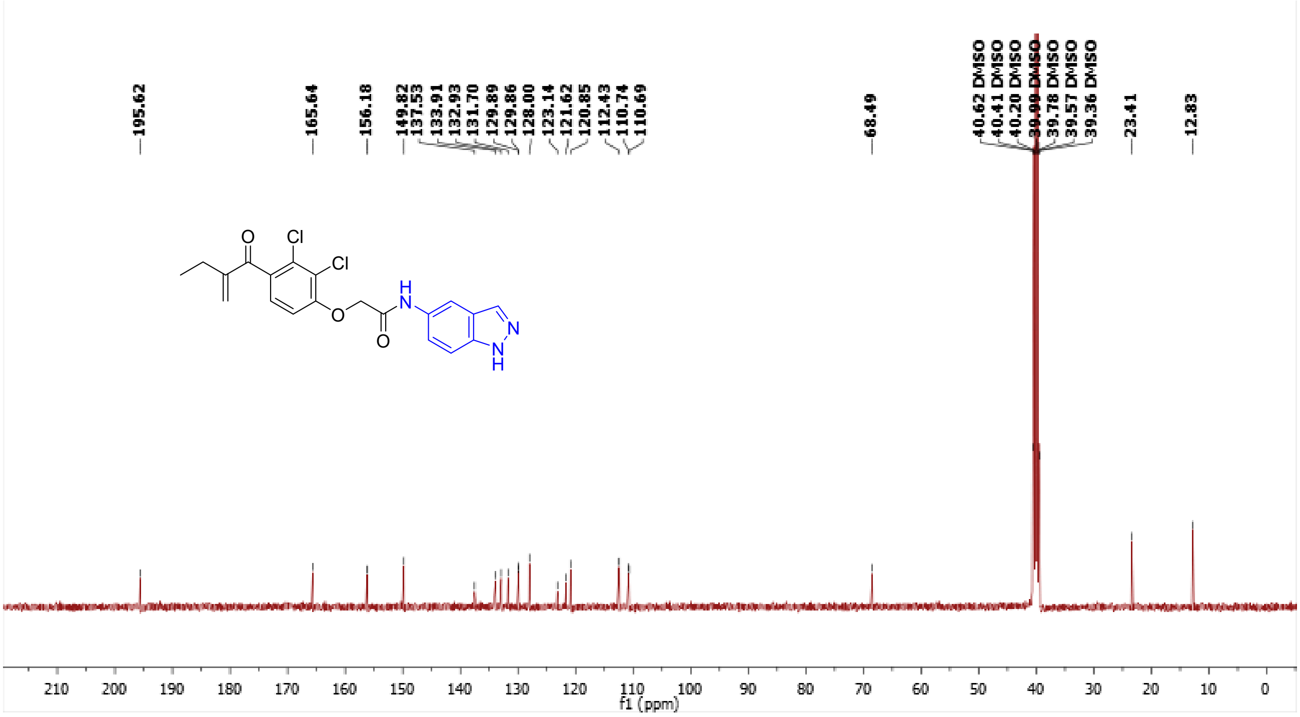

^{13}C NMR spectrum (101 MHz, DMSO) of compound **1**

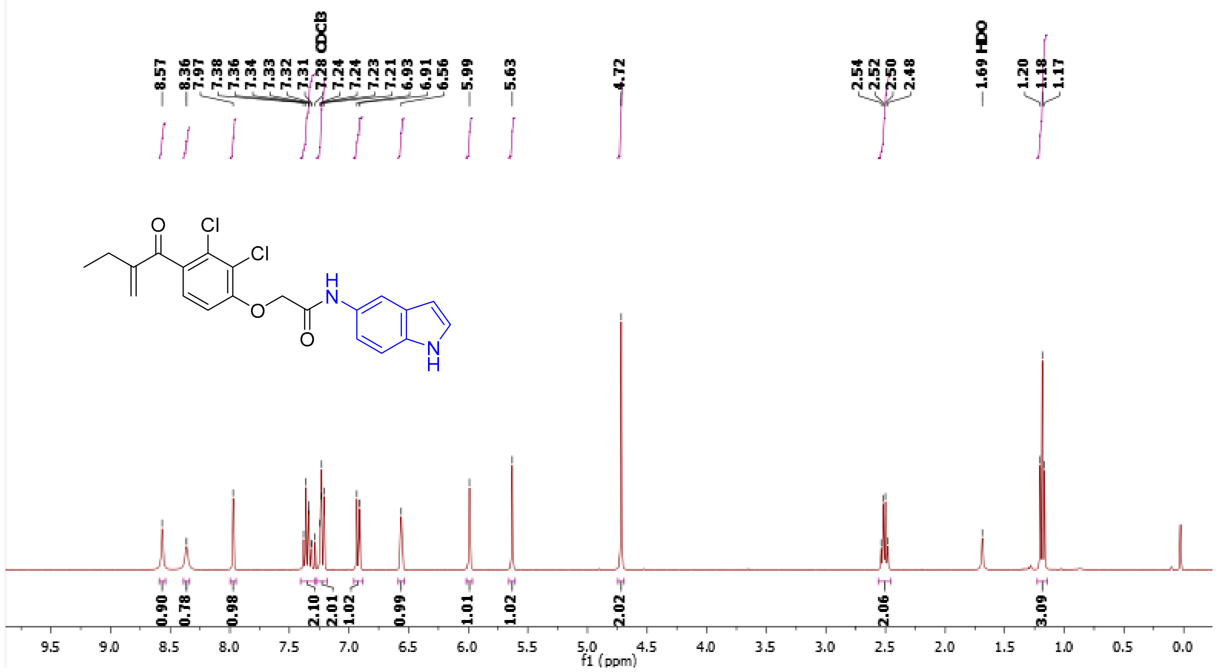

^{1}H NMR spectrum (400 MHz, $CDCl_3$) of compound **2**

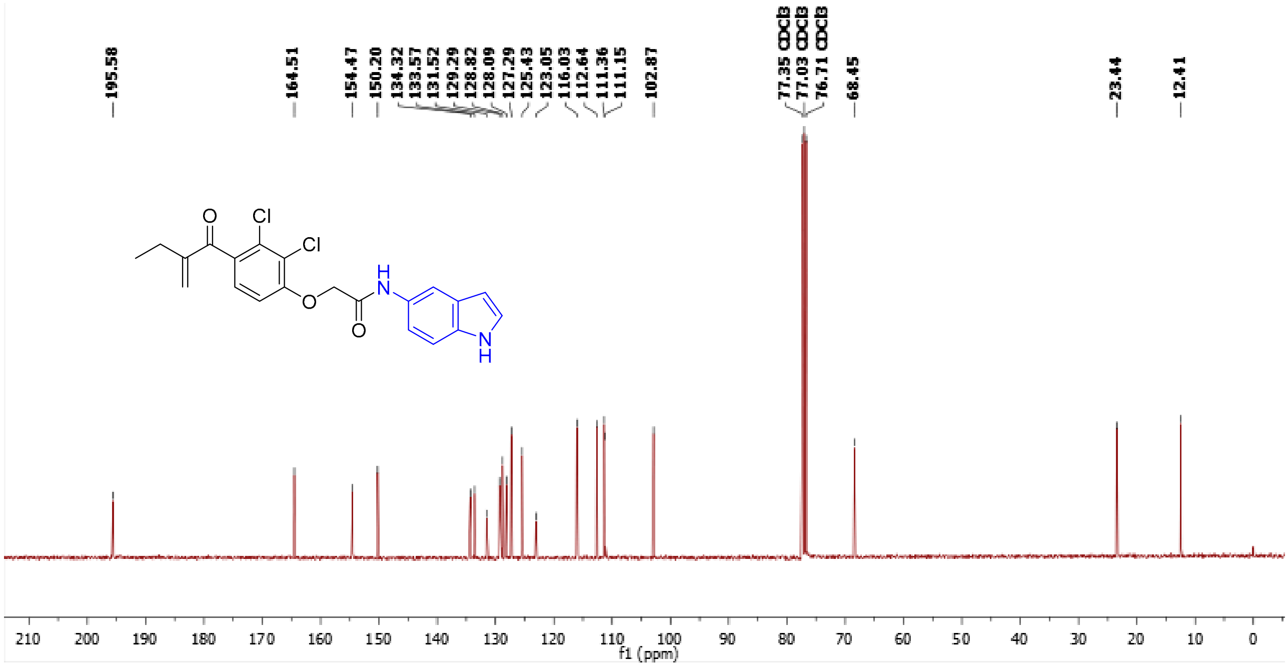

^{13}C NMR spectrum (101 MHz, $CDCl_3$) of compound **2**

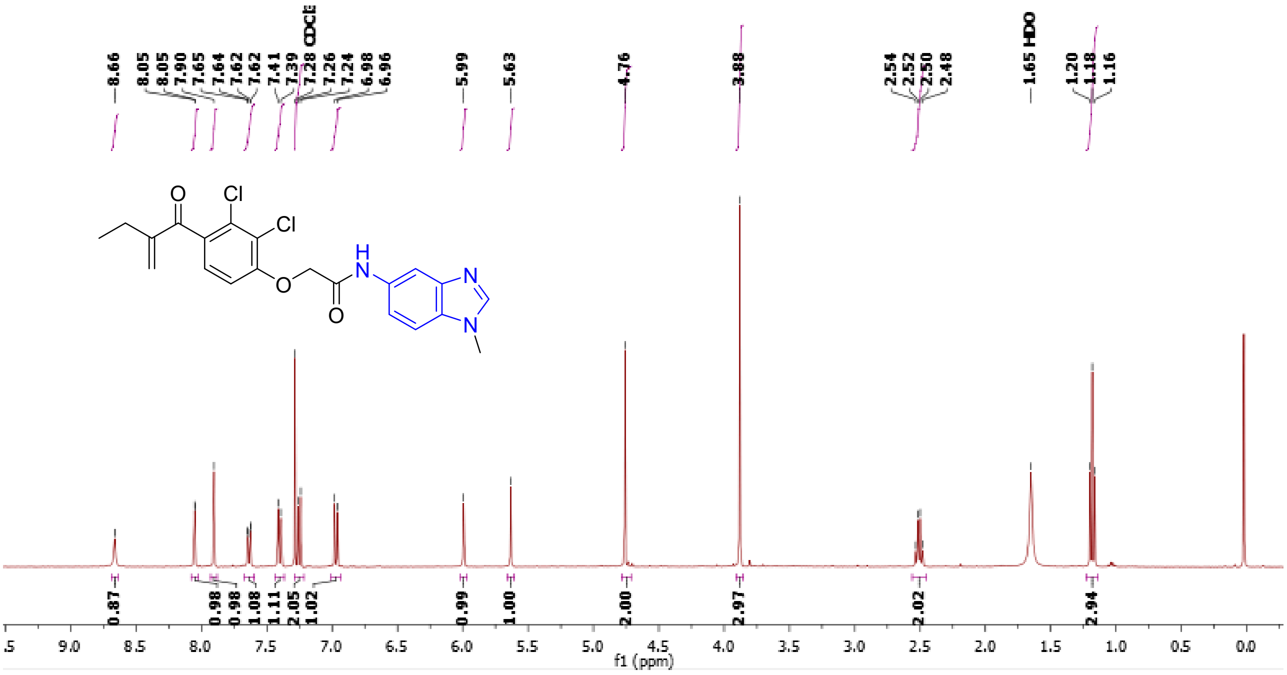

^{1}H NMR spectrum (400 MHz, $CDCl_3$) of compound **3**

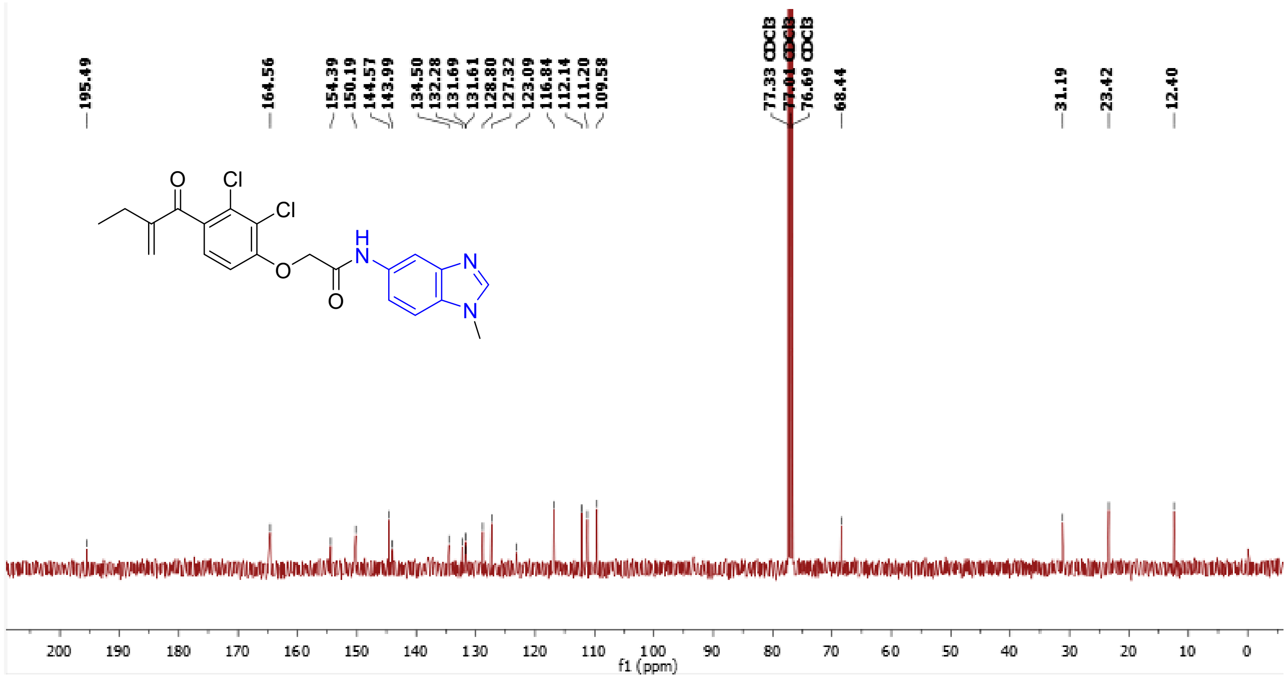

^{13}C NMR spectrum (101 MHz, $CDCl_3$) of compound **3**

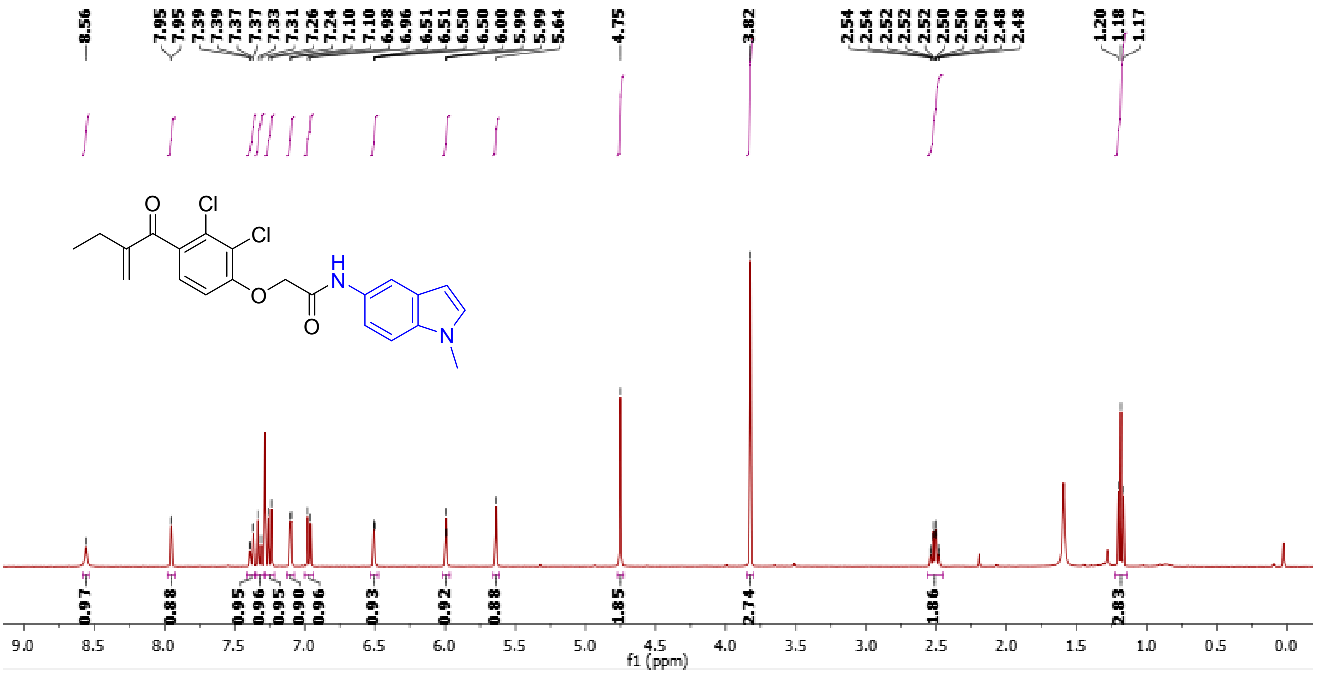

^{1}H NMR spectrum (400 MHz, $CDCl_3$) of compound **4**

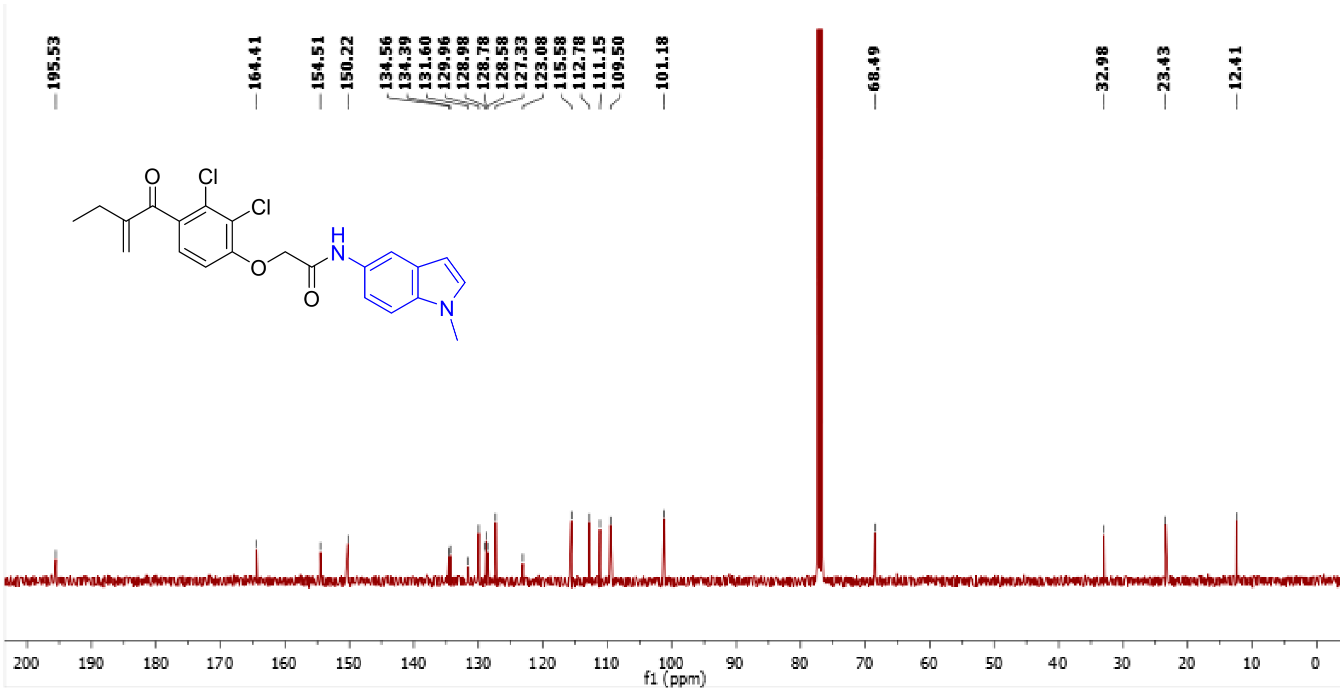

^{13}C NMR spectrum (101 MHz, $CDCl_3$) of compound **4**

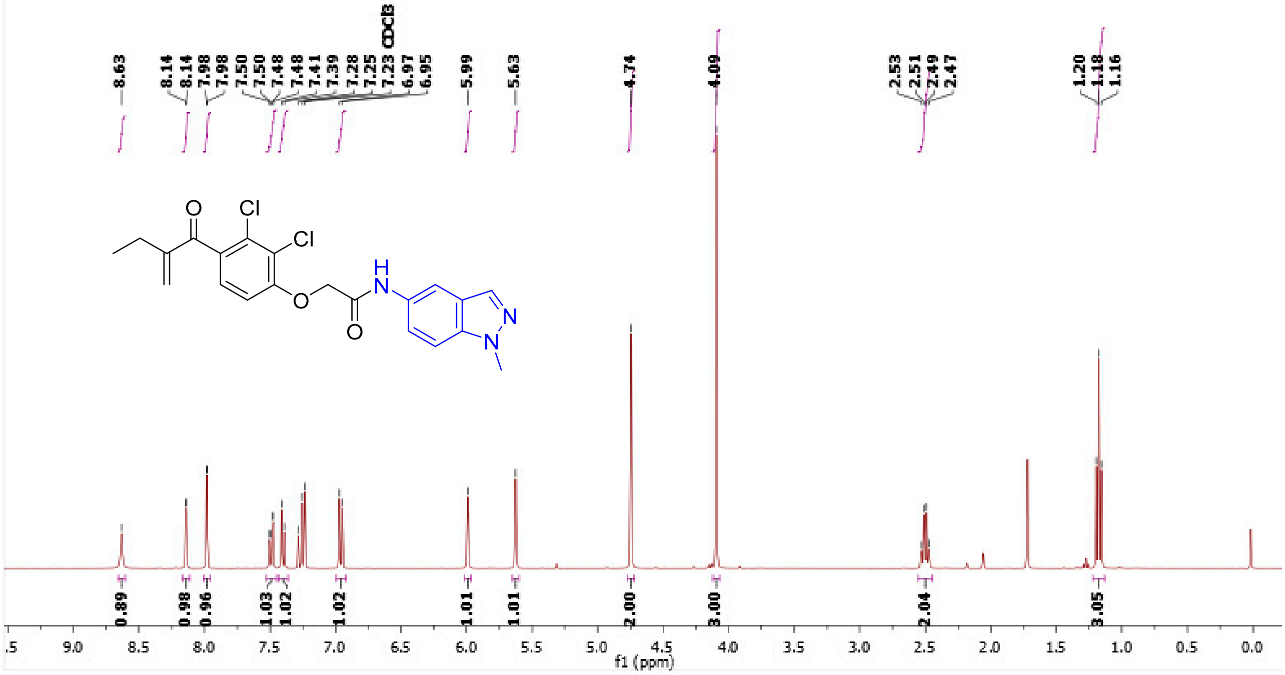

^{1}H NMR spectrum (400 MHz, $CDCl_3$) of compound **5**

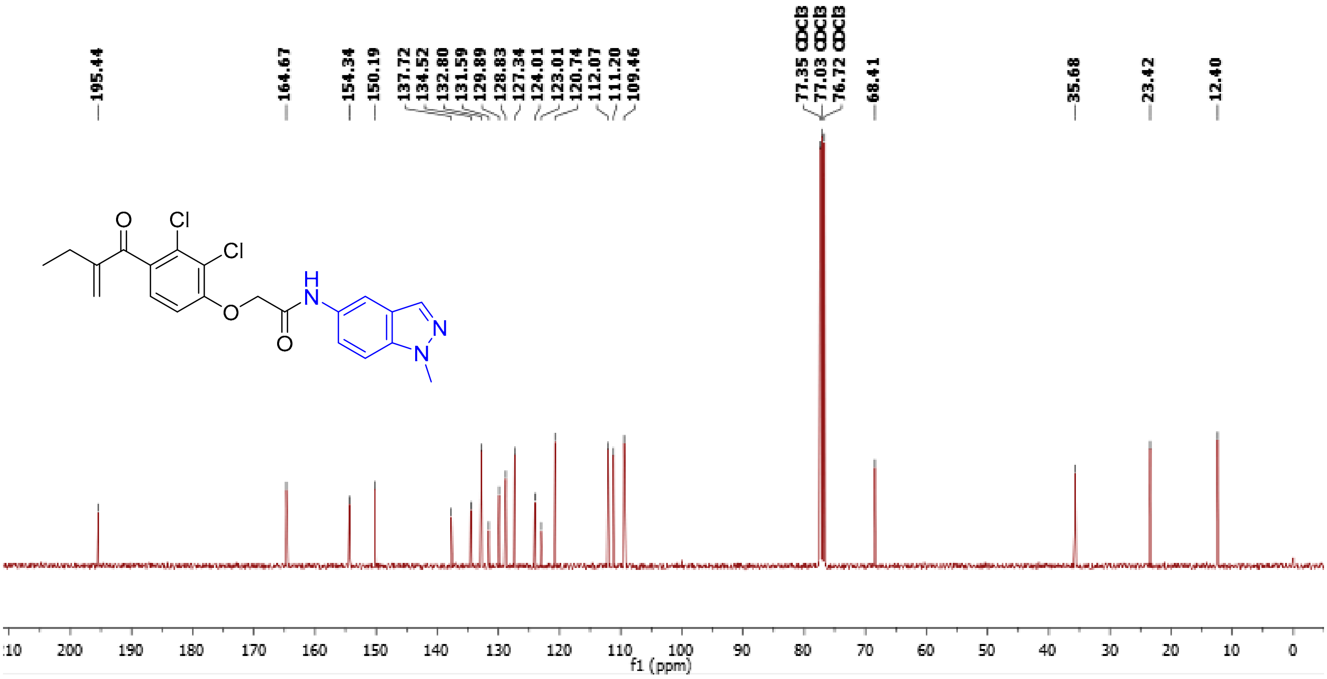

^{13}C NMR spectrum (101 MHz, $CDCl_3$) of compound **5**

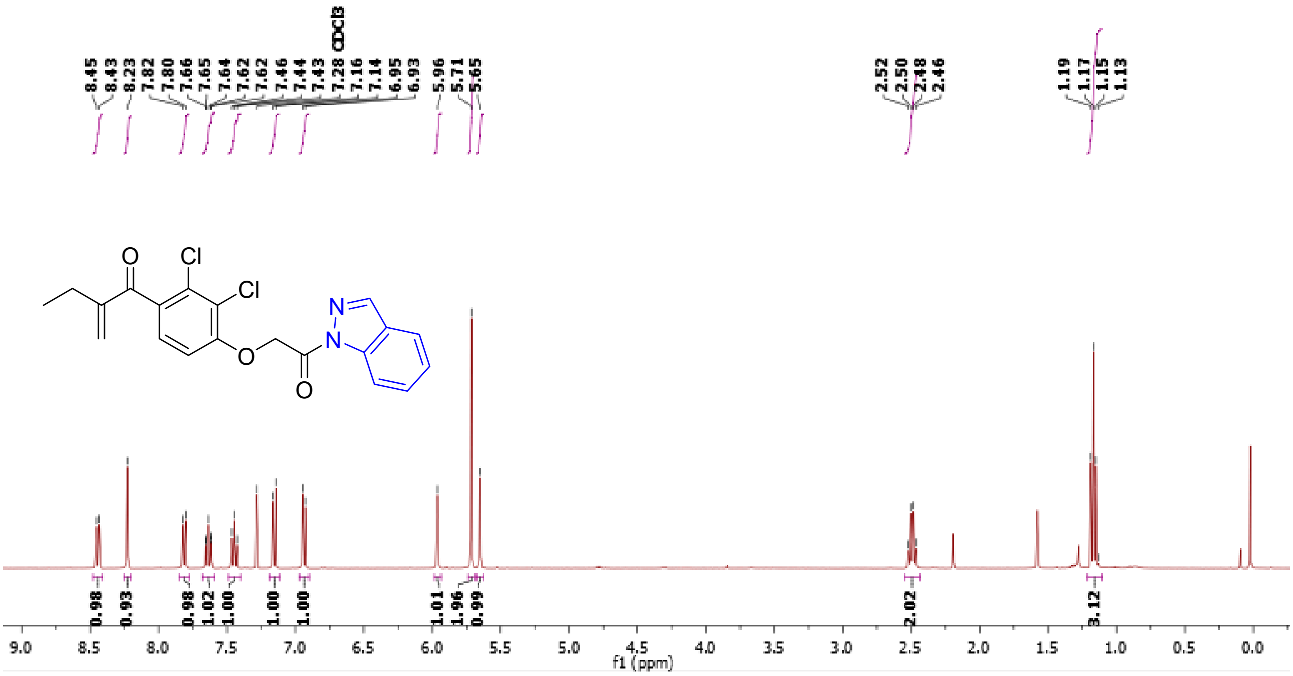

^{1}H NMR spectrum (400 MHz, $CDCl_3$) of compound **6**

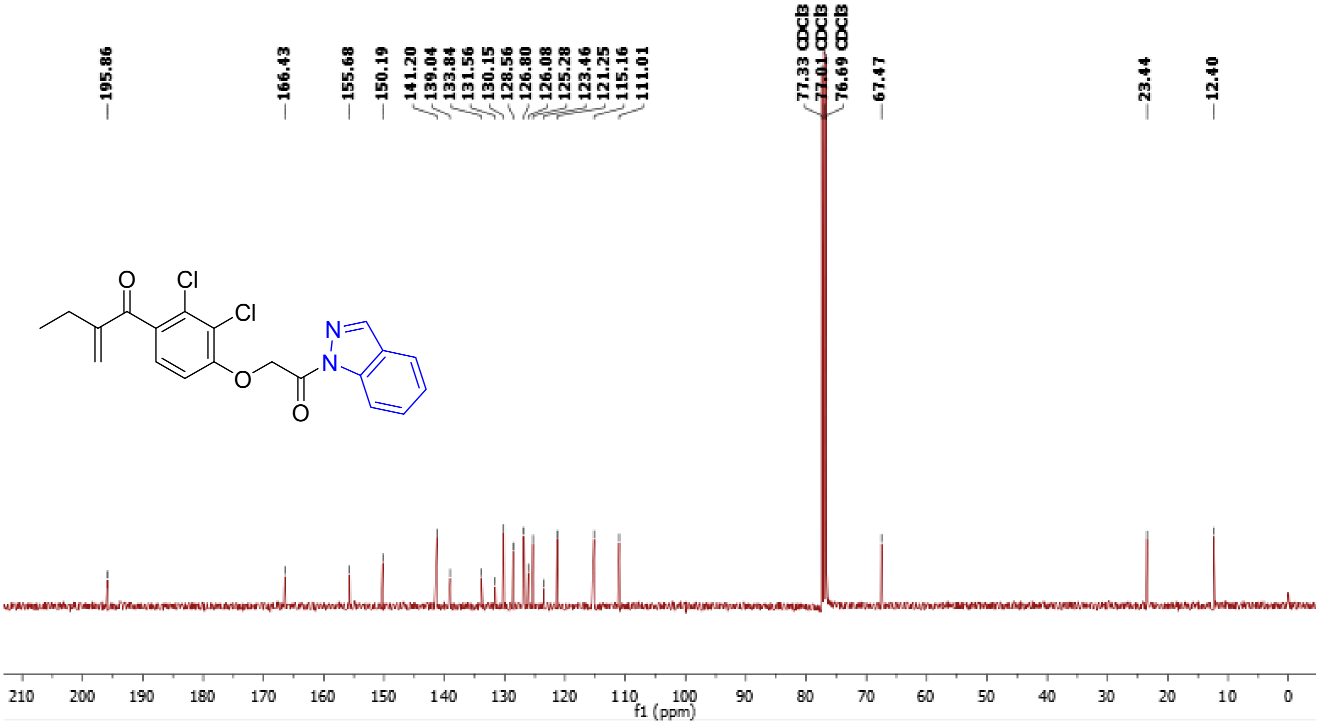

^{13}C NMR spectrum (101 MHz, $CDCl_3$) of compound **6**

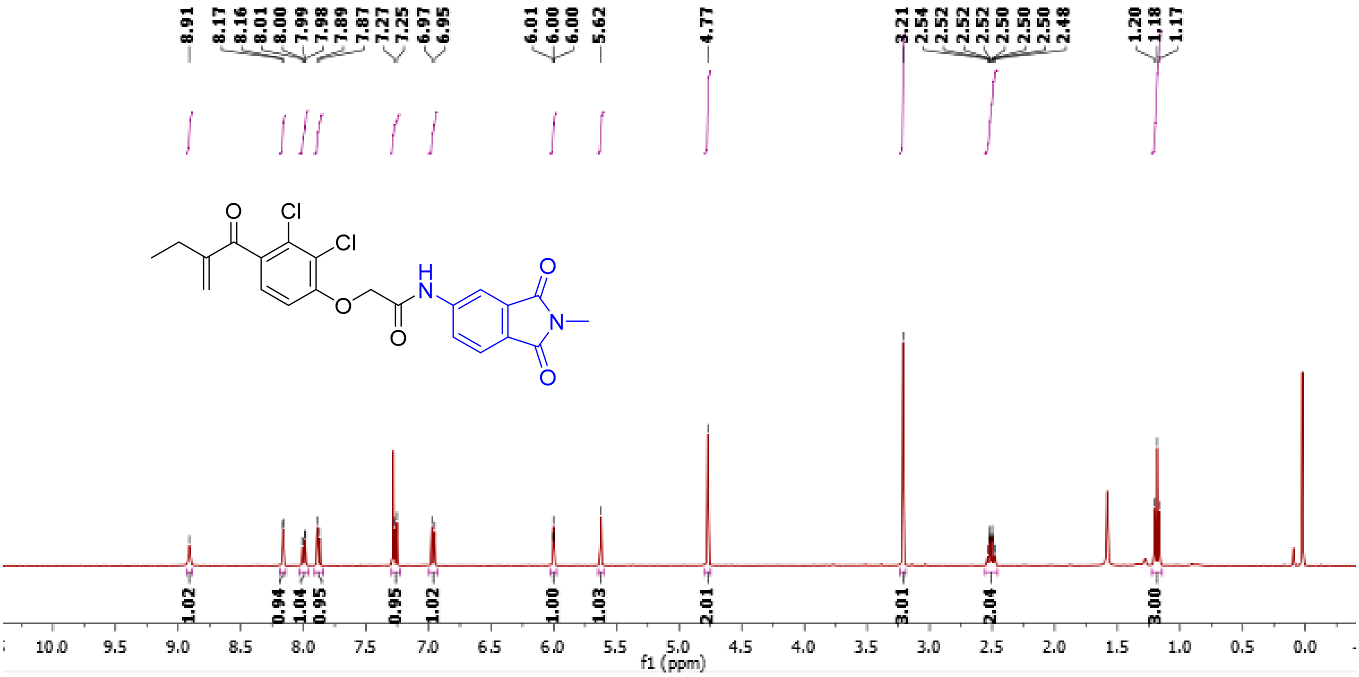

^{1}H NMR spectrum (400 MHz, $CDCl_3$) of compound **7**

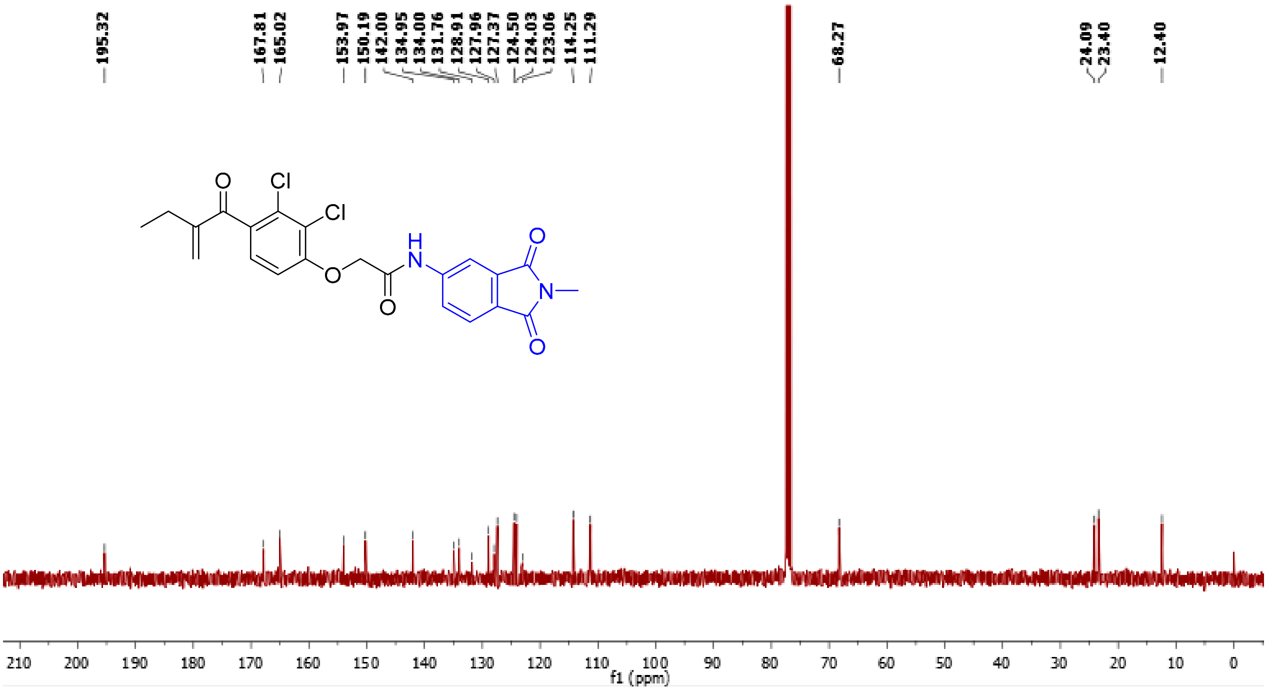

^{13}C NMR spectrum (101 MHz, $CDCl_3$) of compound **7**

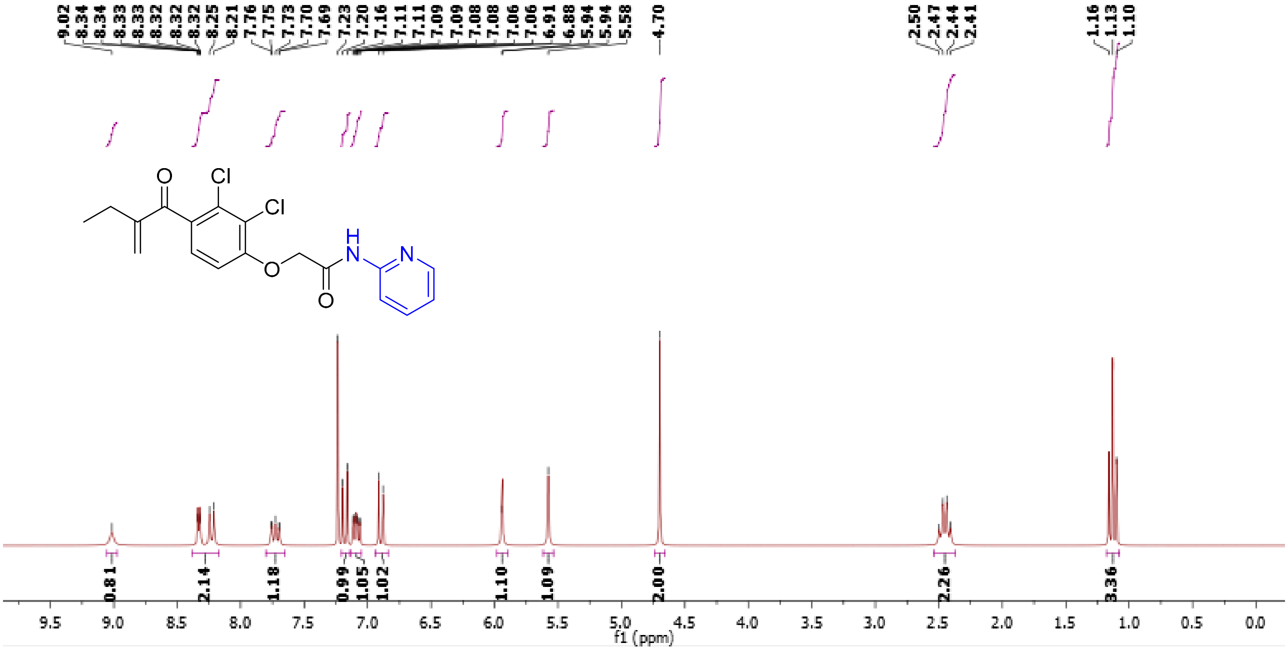

^{1}H NMR spectrum (400 MHz, $CDCl_3$) of compound **8**

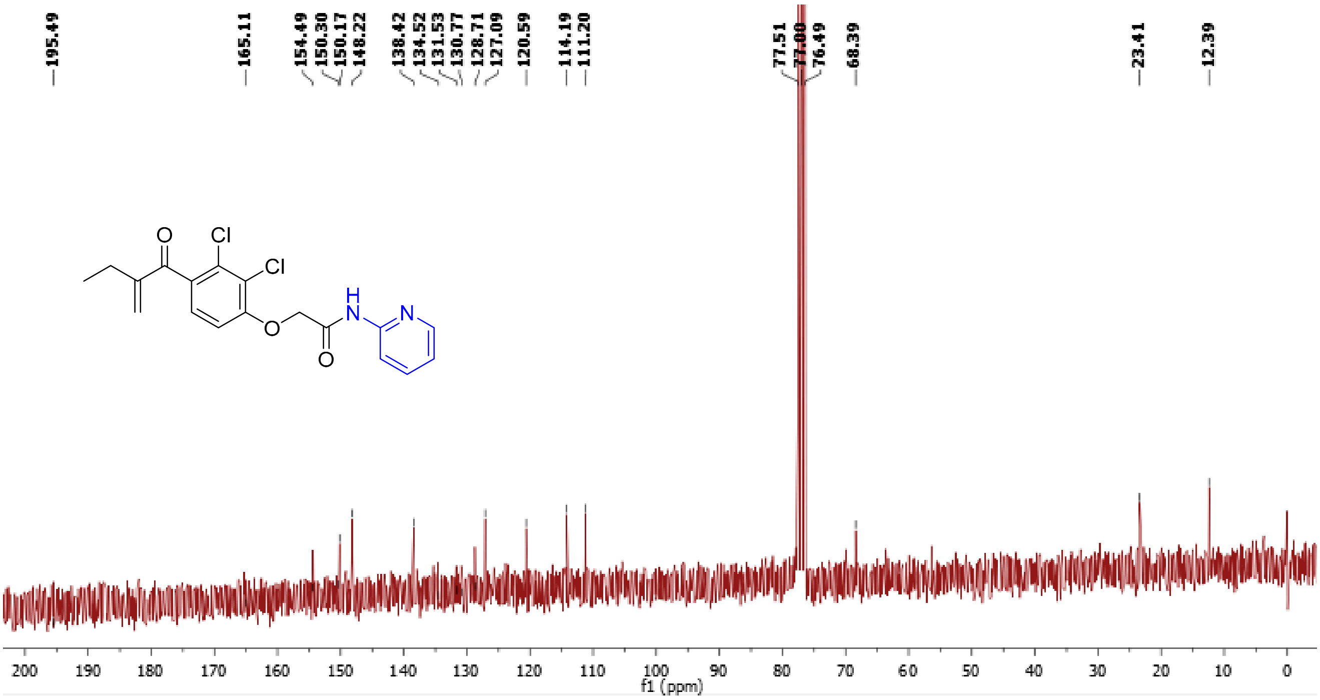

^{13}C NMR spectrum (101 MHz, $CDCl_3$) of compound **8**

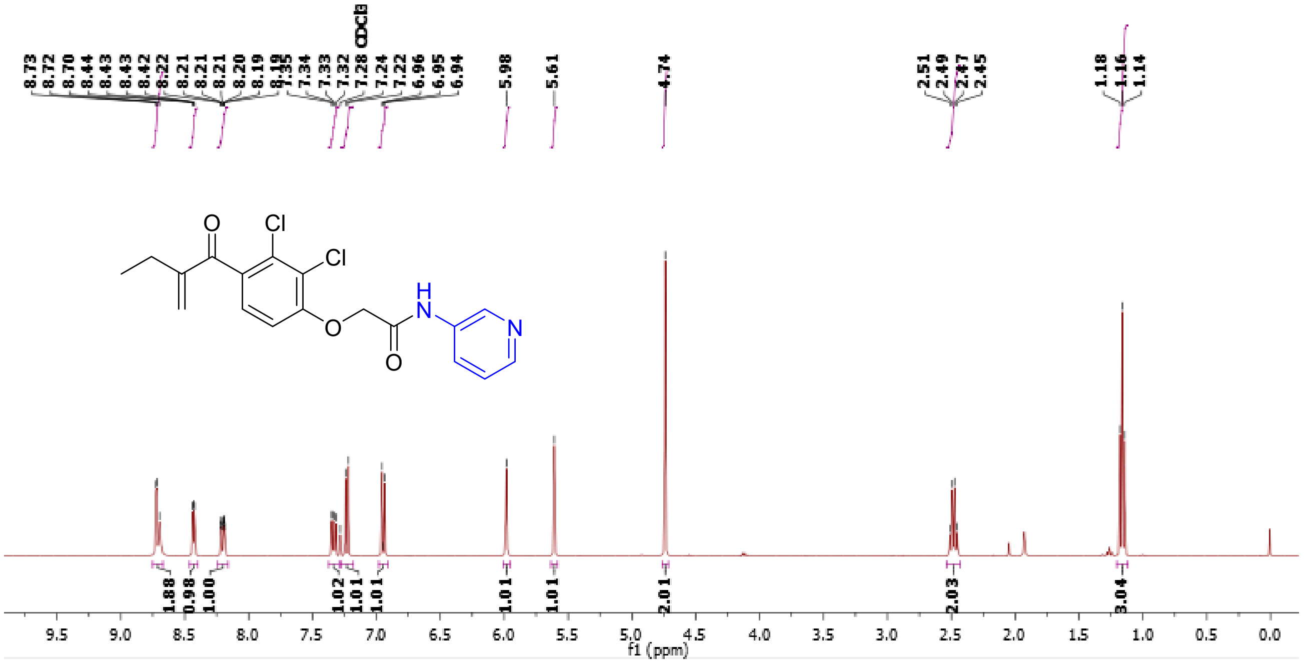

^{1}H NMR spectrum (400 MHz, $CDCl_3$) of compound **9**

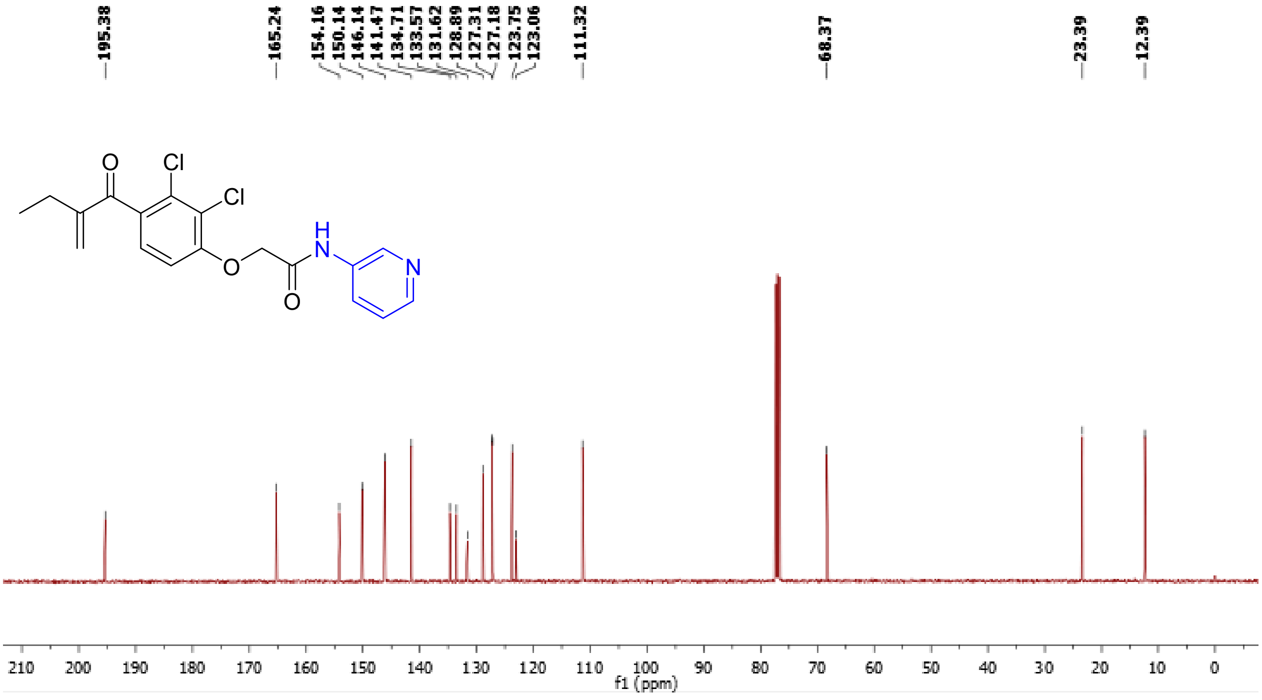

^{13}C NMR spectrum (101 MHz, $CDCl_3$) of compound **9**

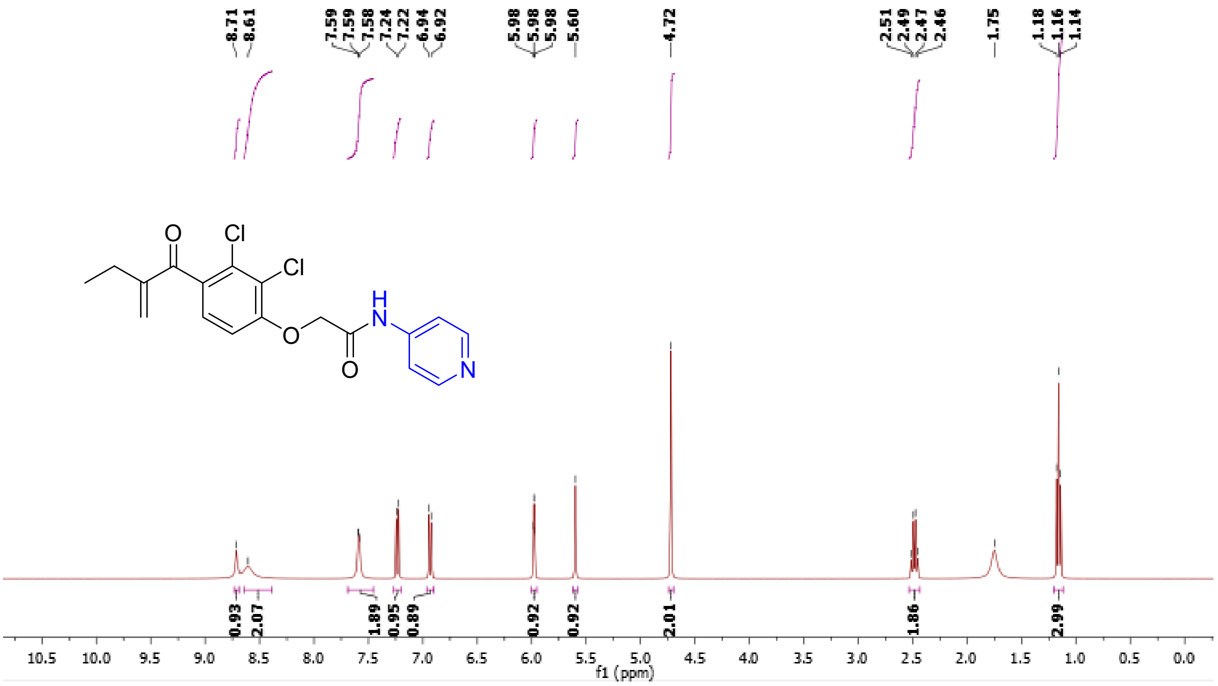

^{1}H NMR spectrum (400 MHz, $CDCl_3$) of compound **10**

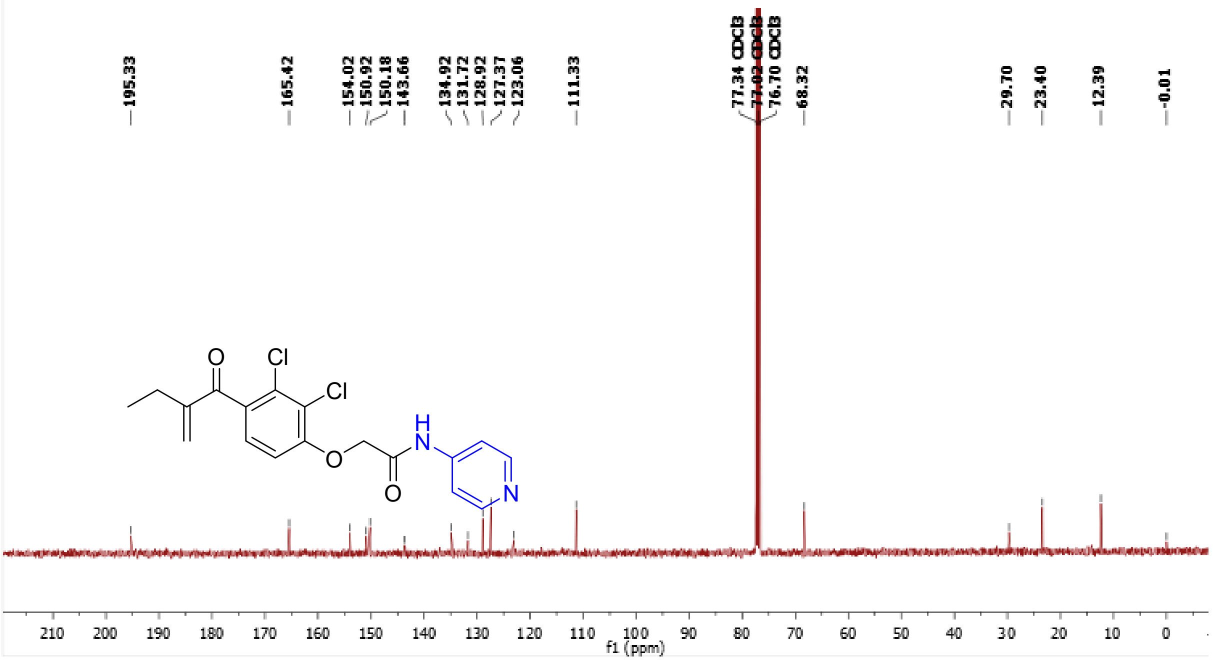

^{13}C NMR spectrum (101 MHz, $CDCl_3$) of compound **10**

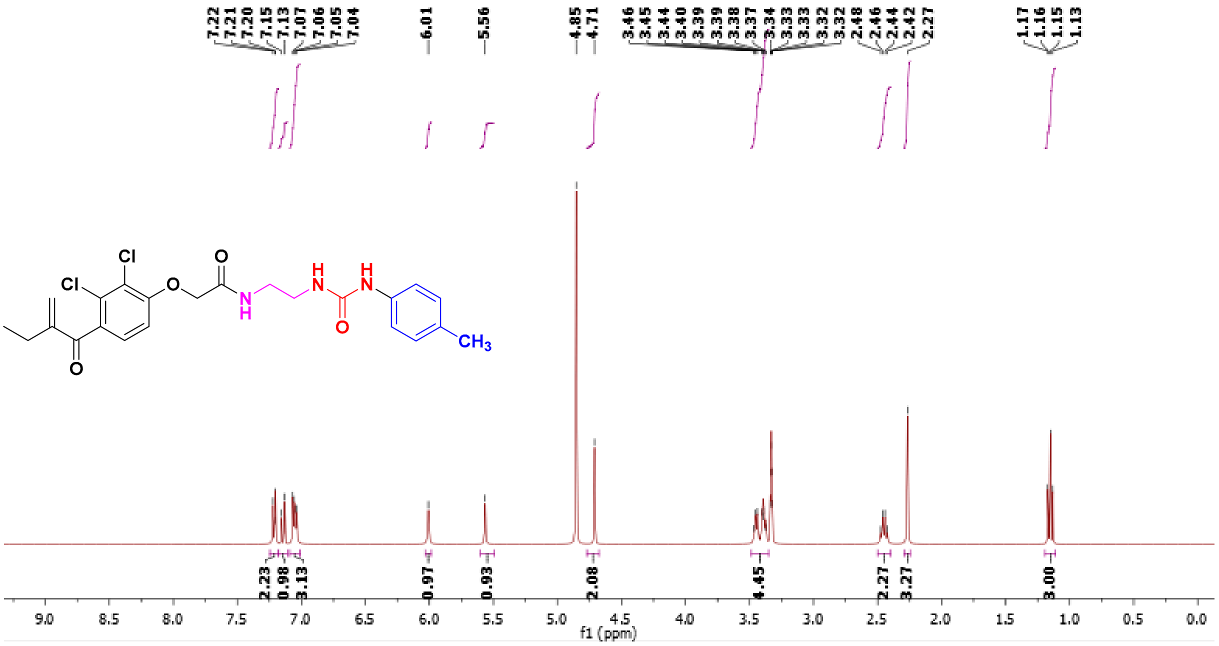

^{1}H NMR spectrum (400 MHz, CD_3OD) of compound **16a**

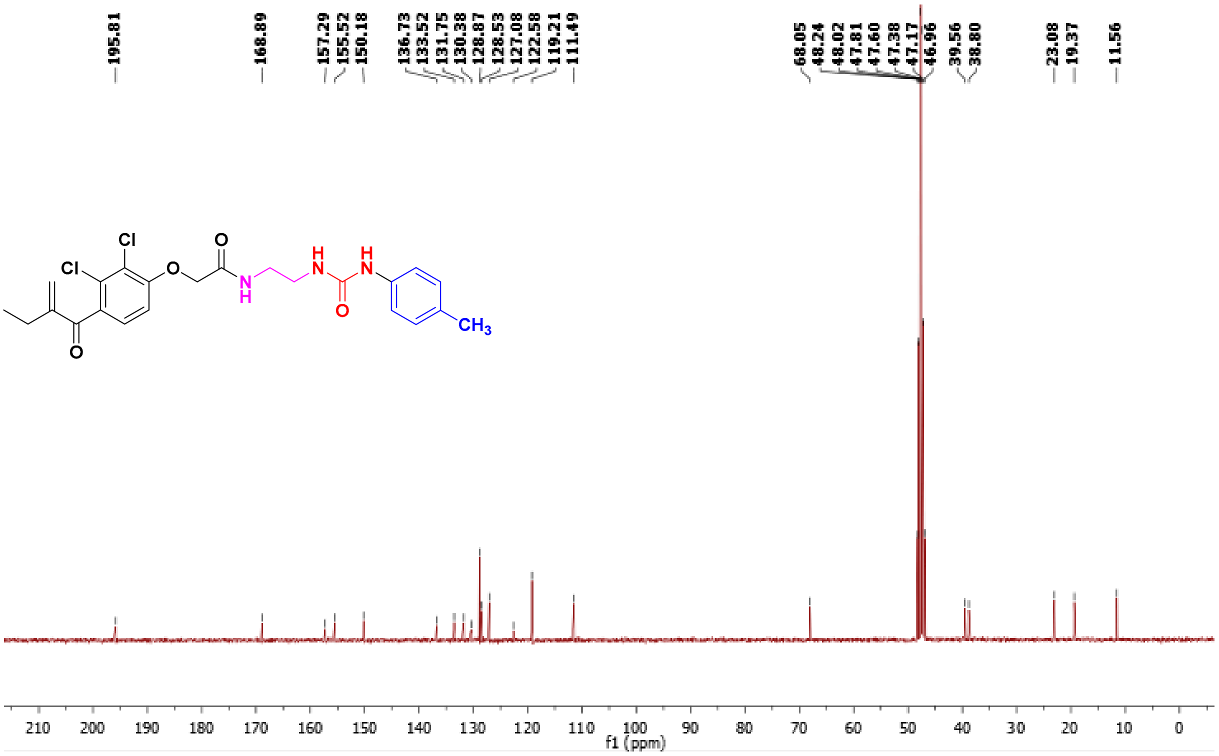

^{13}C NMR spectrum (101 MHz, CD_3OD) of compound **16a**

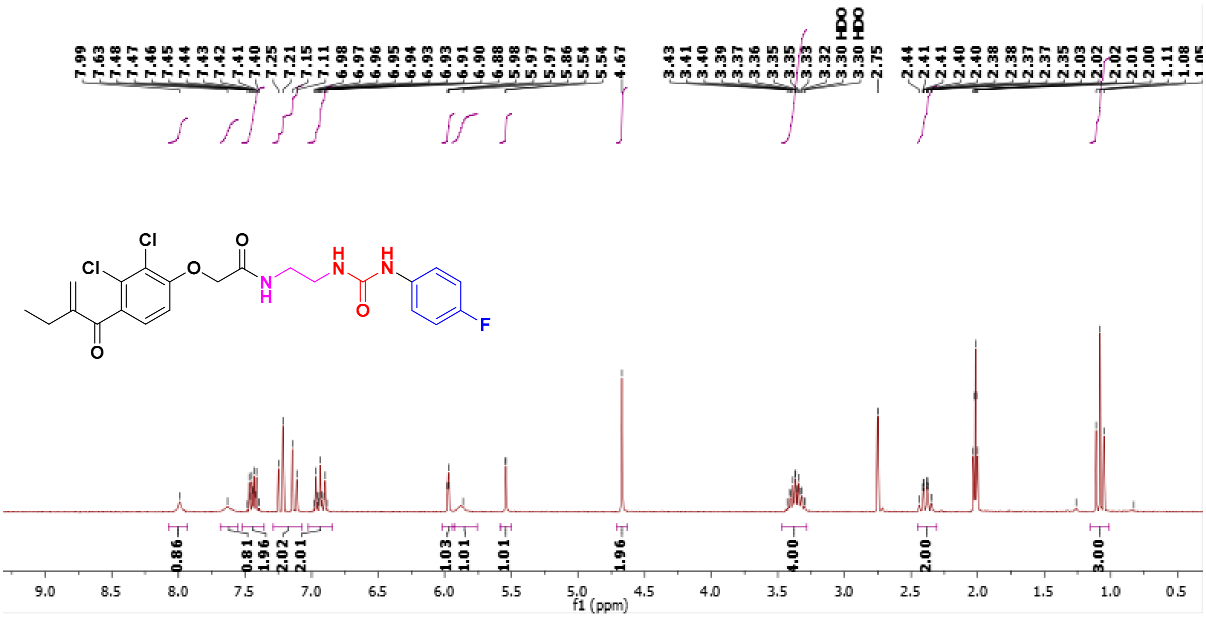

^{1}H NMR spectrum (400 MHz, $(CD_3)_2CO$) of compound **16b**

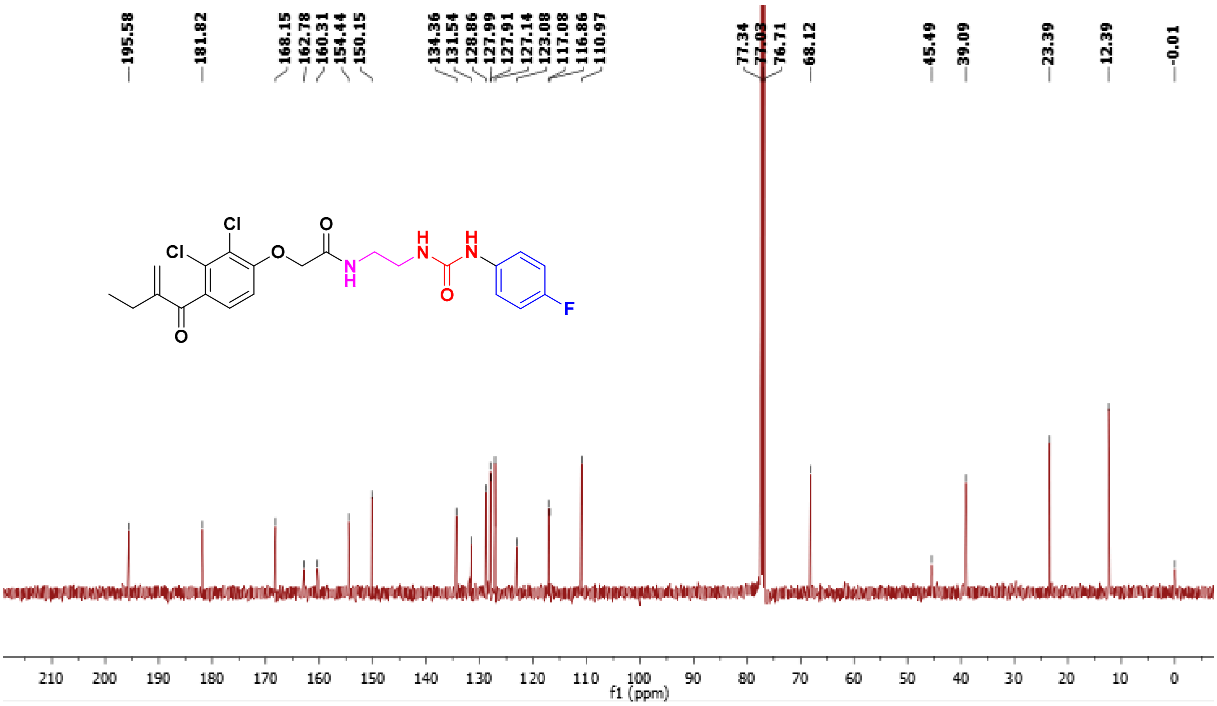

^{13}C NMR spectrum (101 MHz, $CDCl_3$)) of compound **16b**

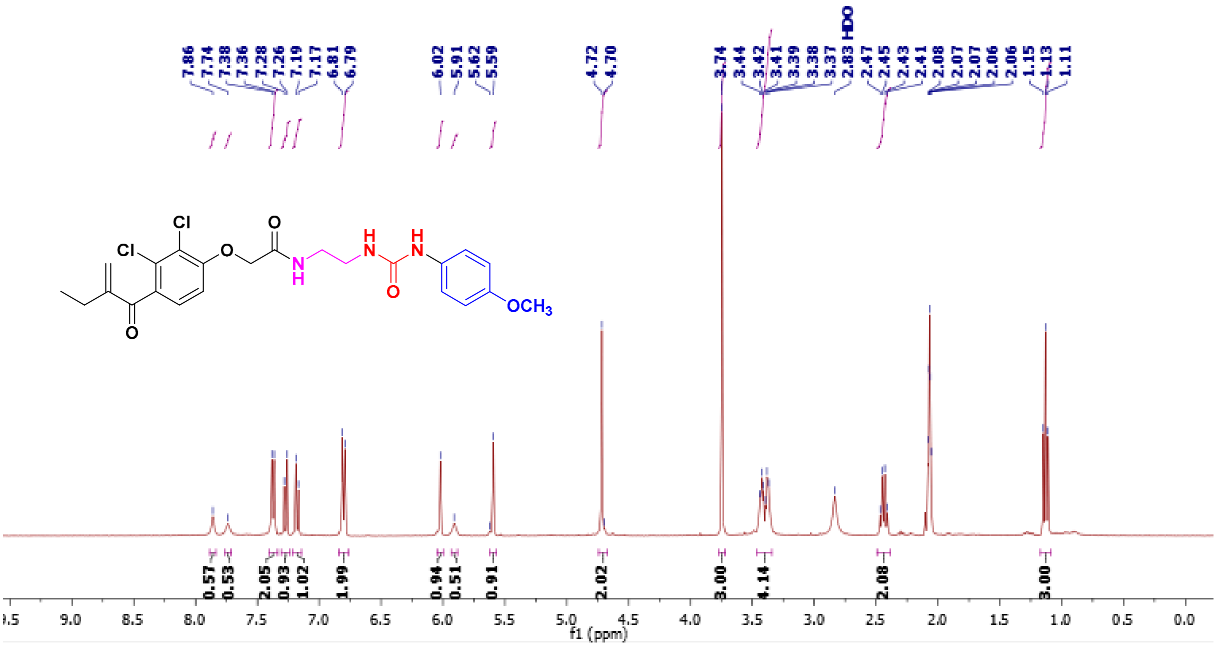

^{1}H NMR spectrum (400 MHz, $(CD_3)_2CO$) of compound **16c**

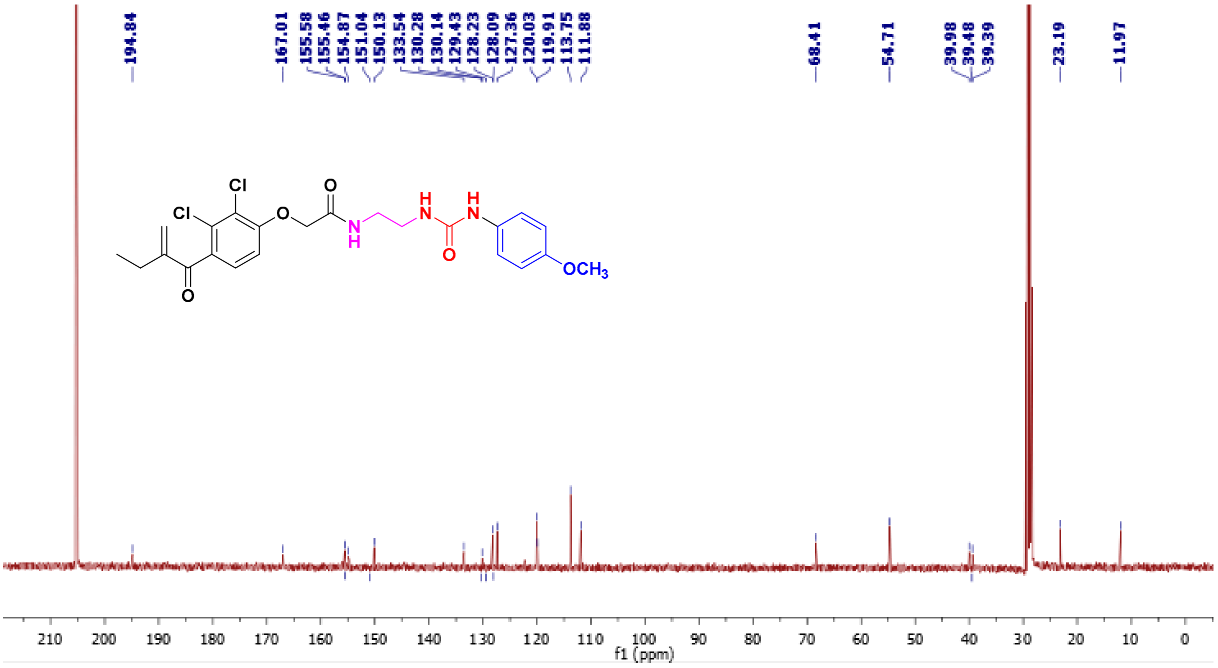

^{13}C NMR spectrum (101 MHz, $(CD_3)_2CO$) of compound **16c**

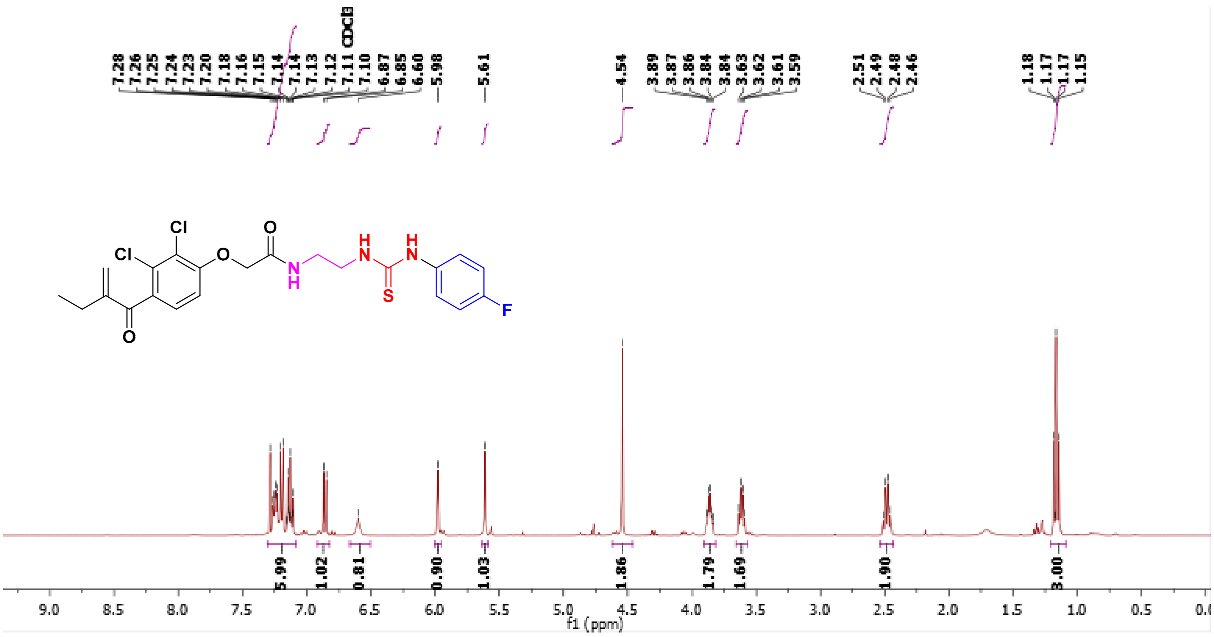

^{1}H NMR spectrum (400 MHz, $CDCl_3$) of compound **17b**

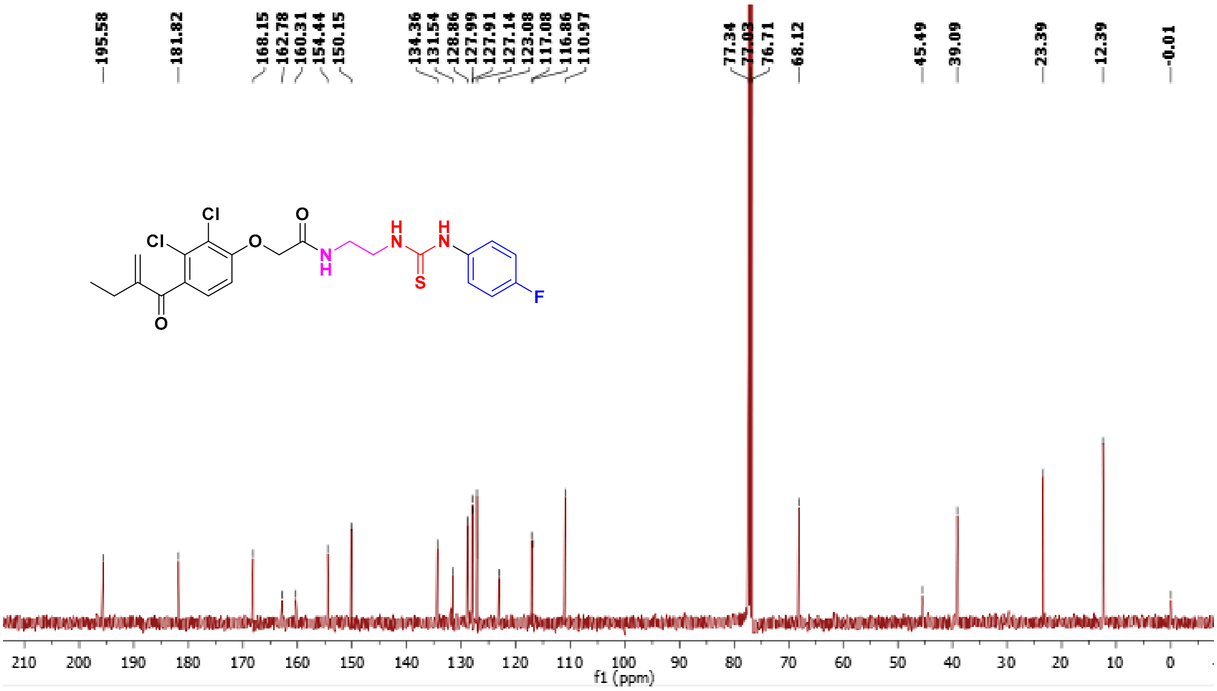

^{13}C NMR spectrum (101 MHz, $CDCl_3$) of compound **17b**

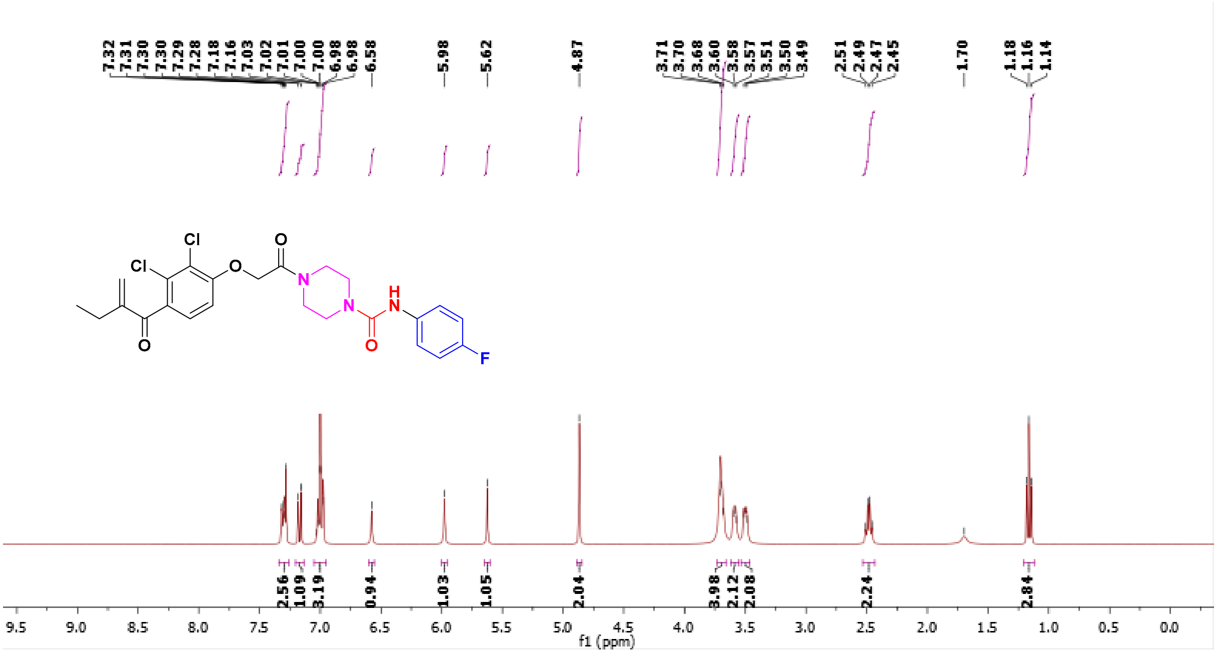

^{1}H NMR spectrum (400 MHz, $CDCl_3$) of compound **18**

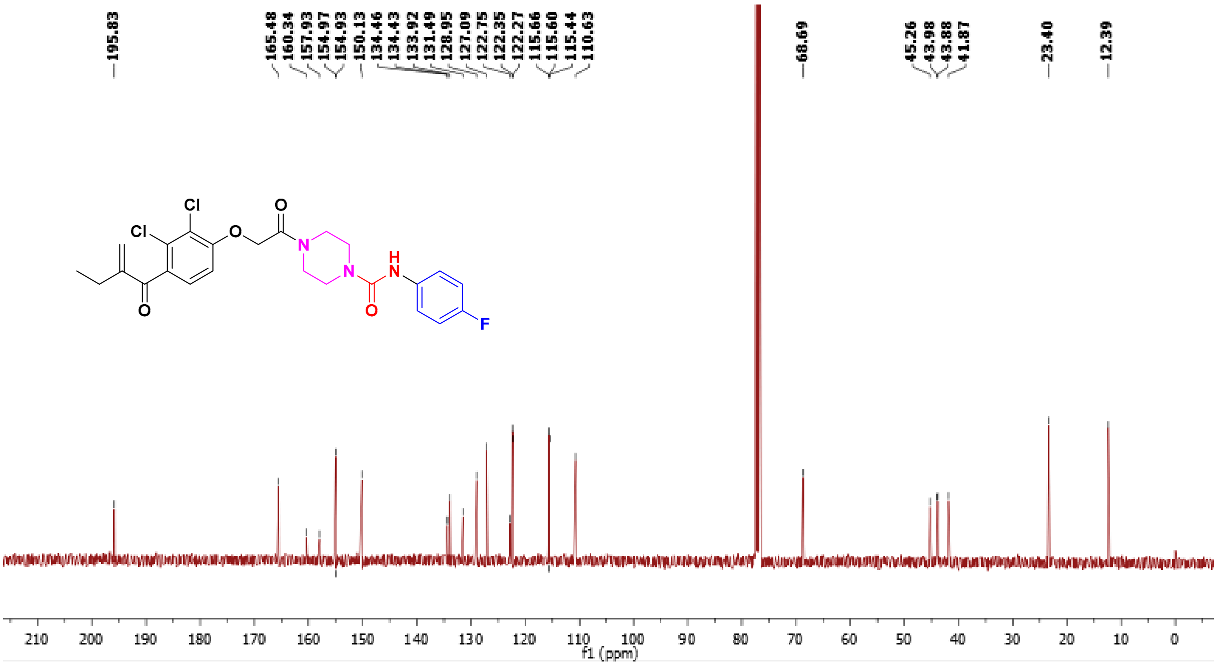

^{13}C NMR spectrum (101 MHz, $CDCl_3$) of compound **18**

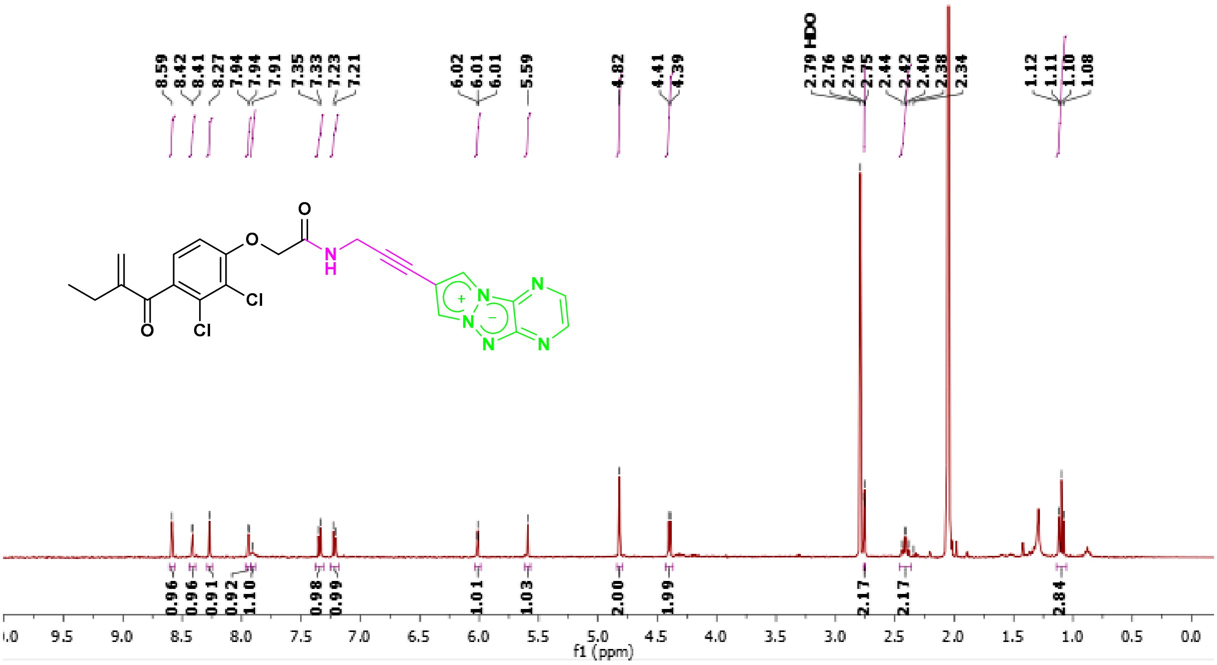

^{1}H NMR spectrum (400 MHz, $(CD_3)_2CO$) of compound **22**

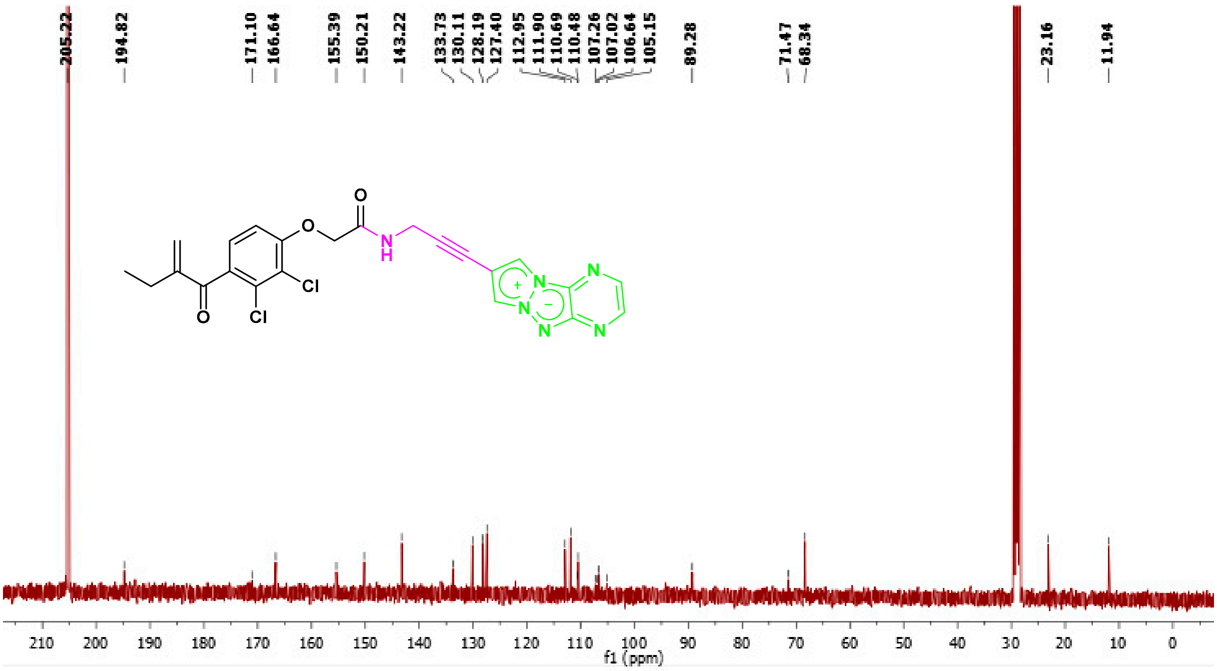

^{13}C NMR spectrum (101 MHz, $(CD_3)_2CO$) of compound **22**

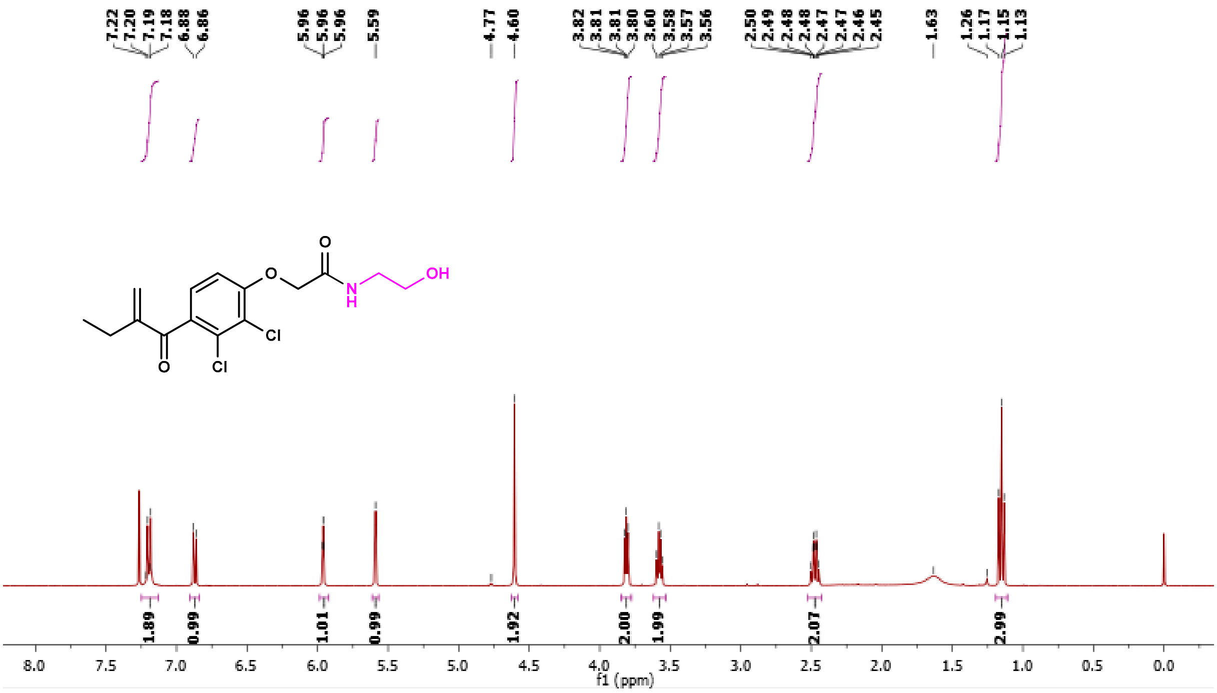

^{1}H NMR spectrum (400 MHz, $CDCl_3$) of compound **23**

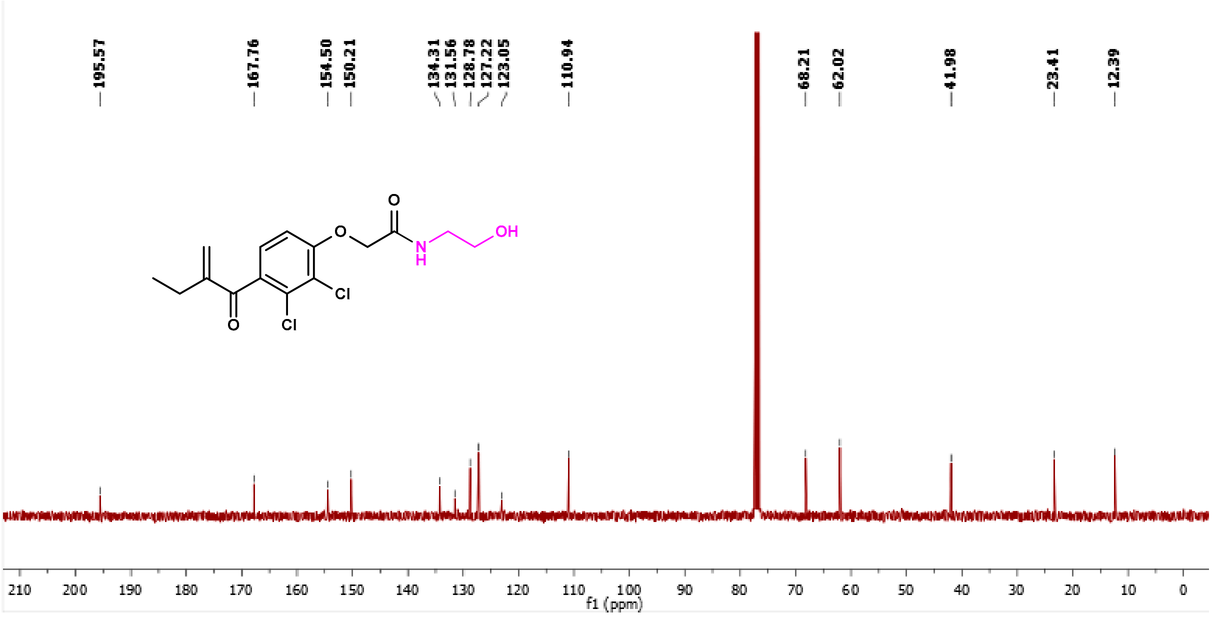

^{13}C NMR spectrum (101 MHz, $CDCl_3$) of compound **23**

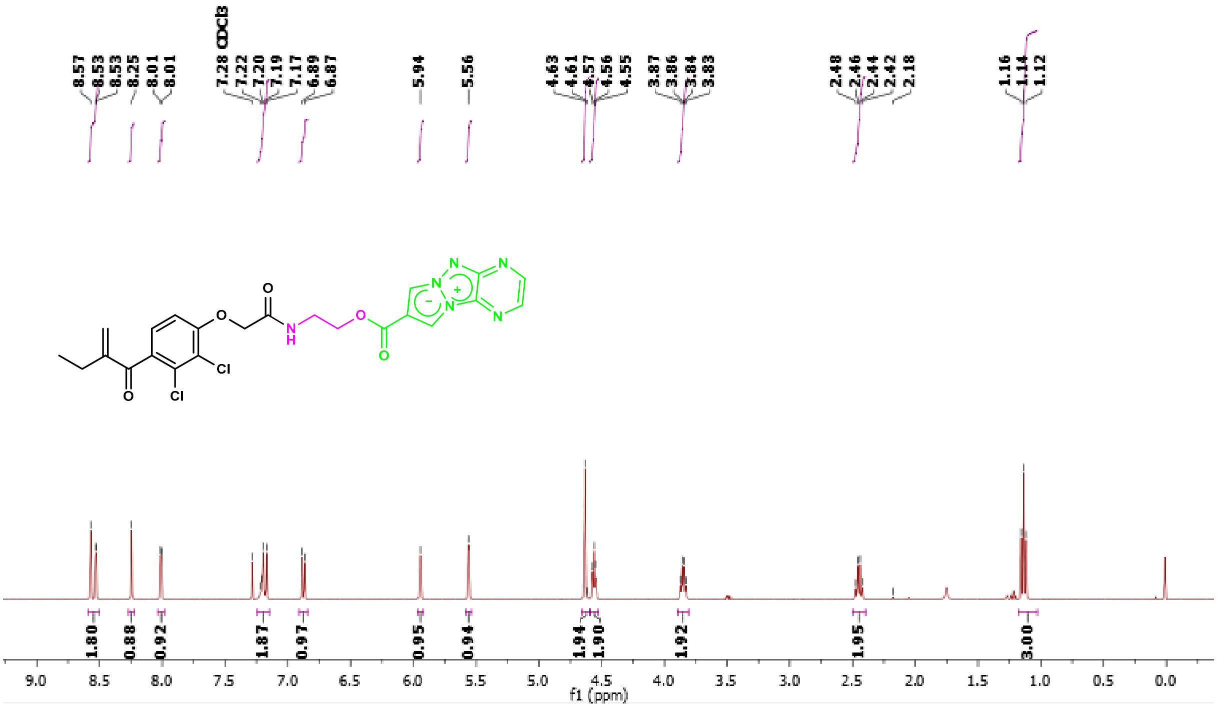

^{1}H NMR spectrum (400 MHz, $CDCl_3$) of compound **24**

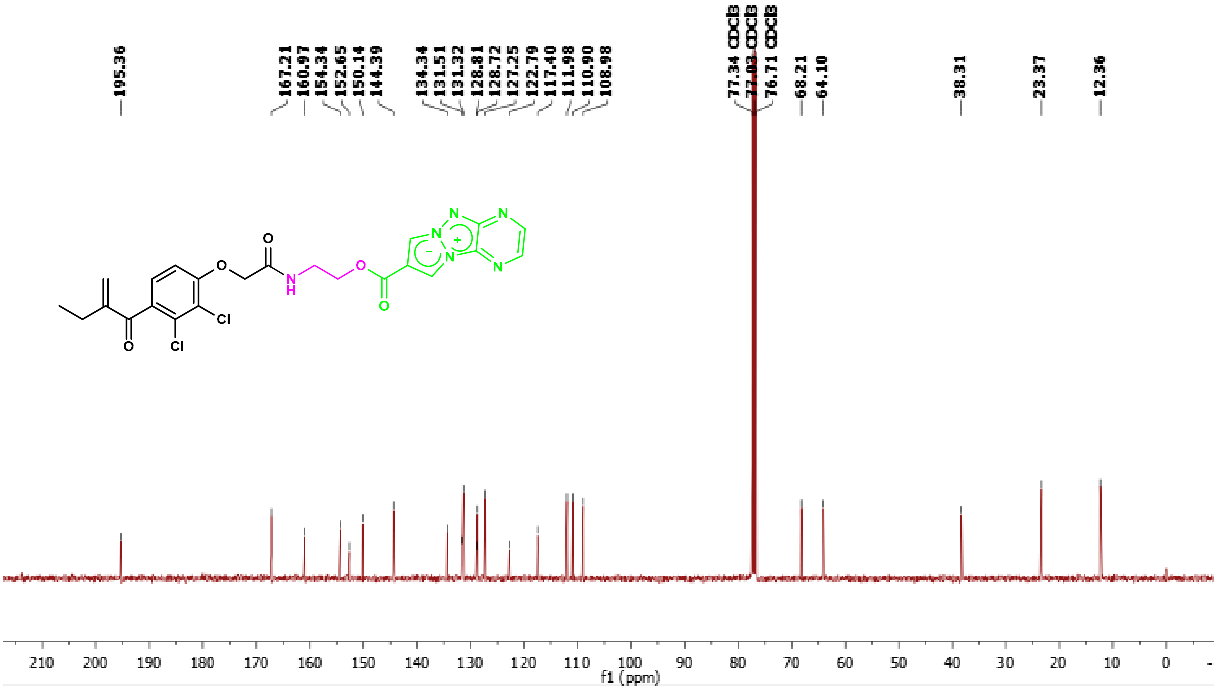

^{13}C NMR spectrum (101 MHz, $CDCl_3$) of compound **24**
